# Evaluation of economic development policies using a spherical fuzzy extended TODIM model with *Z̆*-numbers

**DOI:** 10.1371/journal.pone.0284862

**Published:** 2023-06-13

**Authors:** Shahzaib Ashraf, Muhammad Sohail, Adan Fatima, Sayed M. Eldin

**Affiliations:** 1 Institute of Mathematics, Khwaja Fareed University of Engineering & Information Technology, Rahim Yar Khan, Pakistan; 2 Center of Research, Faculty of Engineering, Future University in Egypt, New Cario, Egypt; Libyan Academy, LIBYA

## Abstract

Zadeh’s *Z̆*-numbers are able to more effectively characterize uncertain information. Combined with “constraint” and “reliability”. It is more powerful at expressing human knowledge. While the reliability of data can have a direct impact on the precision of decisions. The key challenge in solving a *Z̆*-number issue is reasoning about both fuzzy and probabilistic uncertainty. Existing research on the *Z̆*-number measure is only some, and most studies cannot adequately convey the benefits of *Z̆*-information and the properties of *Z̆*-number. Considering this study void, this work concurrently investigated the randomness and fuzziness of *Z̆*-number with Spherical fuzzy sets. We first introduced the spherical fuzzy Z-numbers (SFZNs), whose elements are pairwise comparisons of the decision-maker’s options. It can be used effectively to make true ambiguous judgments, reflecting the fuzzy nature, flexibility, and applicability of decision making data. We developed the operational laws and aggregation operators such as the weighted averaging operator, the ordered weighted averaging operator, the hybrid averaging operator, the weighted geometric operator, the ordered weighted geometric operator, and the hybrid geometric operator for SF*Z̆*Ns. Furthermore, two algorithm are developed to tackle the uncertain information in the form of spherical fuzzy *Z̆*-numbers based to the proposed aggregation operators and TODIM methodology. Finally, we developed the relative comparison and discussion analysis to show the practicability and efficacy of the suggested operators and approach.

## 1 Introduction

In real life, systems get more complicated every day, making it hard for people in charge to choose the best option from a variety of choices. It’s challenging to explain, but not impossible, to reach a certain goal. Setting motivation, objectives, and perspectives are complexities that many firms find challenging. So, whether a person or a committee makes a decision, they must simultaneously consider several goals. This theory suggests that criteria are flexible, which prohibits any decision maker from attaining the optimal answer for each criterion involved in the actual circumstance. As a consequence, the decision-maker is more concerned with establishing approaches that are more suitable and proficient for identifying the optimal choice. In dealing with ambiguous and unclear facts in decision-making situations, the classical or crisp approach may not always be effective.

Spherical fuzzy sets are a type of fuzzy set that allows for a more flexible representation of uncertainty in a multidimensional space. In a traditional fuzzy set, each element is assigned a degree of membership between 0 and 1, representing the degree to which the element belongs to the set. In contrast, spherical fuzzy sets assign a degree of membership to a subset of a multidimensional space, rather than to individual elements. The subset is defined by a center point and a radius, representing the degree of membership of all elements within a certain distance from the center point. The degree of membership for an element is determined by its distance from the center point, with elements closer to the center point having a higher degree of membership. The radius of the spherical fuzzy set determines the degree of uncertainty or fuzziness around the center point. Spherical fuzzy sets have applications in fields such as decision making, data mining, and pattern recognition, where uncertainty in multidimensional data needs to be taken into account.

In 1965, in response to this unpredictability, Zadeh [1]assigned a membership grade ranging from zero to one to each individual component of a set. Fuzzy sets have many of the set-theoretic properties of crisp circumstances. Zadeh’s work in this area is impressive. FSs can be used in different ways in decision science, communications, medical science, intelligence science, marketing, engineering, computer science, and other fields. Banerjee et al. [2] provided a comprehensive review of *Z̆*-numbers, a type of uncertain number that extends the concept of fuzzy numbers. The authors highlighted the unique features and advantages of *Z̆*-numbers, such as their ability to handle both randomness and fuzziness in a unified framework, and their ability to capture a wide range of uncertainty. The review also discussed the applications of *Z̆*-numbers in various fields, such as decision-making, pattern recognition, and control systems. Overall, the review provided valuable insights into the theory and applications of *Z̆*-numbers, making it a useful resource for researchers and practitioners in the field of uncertainty modeling.

The fuzzy number, a useful tool in uncertain situations, can reflect human judgment, but it ignores the rationality of the information, which is crucial for planning, making decisions, creating algorithms, and managing information. As a result, Zadeh [3] tackles such types of limitations with a fuzzy *Z̆*-number which provides an explanation for the limitation as well as the precision of the judgement. *Z̆* = (Y, W) is an ordered pair of fuzzy numbers, where Y is the fuzziness constraint on the value of variable N and W is a measure of assurance or other relevant notions like certainty, confidence, dependability, likelihood, etc. It looks more adaptable and significant from an intuitive perspective for formalizing the functionality of a decision-making procedure. Many scholars work with fuzzy *Z̆*- numbers such as ranking *Z̆*-numbers [4], numerical solution of fuzzy equation [5], modeling for uncertain nonlinear systems [6], Hukuhara difference [7], decision-making using *Z̆*-numbers [8, 9],etc.

Atanassov’s [10] work on intuitionistic fuzzy set (IFS) was extremely impressive because he expanded the idea of FS by allocating membership degrees as *α*(*n*) together with a non-membership degree as *β*(*n*) with the constraint that 0 ≤ *α*(*n*) + *β*(*n*)≤1. Although Atanassov’s creation of IFSs is highly regarded, although decision-makers are somewhat constrained in determining degrees due to the constraint of *α*(*n*) and *β*(*n*). Sometimes the total of combined membership degrees exceeds 1. IFS fails in this circumstance to produce a reasonable result. As a result, Yager [11] established the notion of Pythagorean fuzzy sets (PFS) in order to cope with this scenario. He did this by designating membership degrees, as *α*(*n*) along with non-membership degrees, as *β*(*n*) with the restriction that 0 ≤ *α*^2^(*n*) + *β*^2^(*n*) ≤ 1.

Atanassov’s structure only discusses the degree of satisfaction and dissatisfaction among a group of elements, which is rather inadequate given that human nature also includes concerns with reluctance and abstention. Such obstacles were taken into account by Cuong [12] when he proposed picture fuzzy sets (P-FS), which he defined as (*α*(*n*), *γ*(*n*), and *β*(*n*)), where each component upon the triplet serves as satisfaction, indeterminacy, and dissatisfaction degrees, respectively. This was subject to the provision that 0 ≤ *α*(*n*) + *γ*(*n*) + *β*(*n*)≤1. Compared to past notions, the Cuong structure is closer to human nature and was one of the most abundant research areas. In 2014, Cuong [13] established relationships, compositions, and distance measurements between image fuzzy numbers. Phong [14] provided an idea regarding the compositions of various picture fuzzy relations.

Although Cuong’s creation of picture fuzzy sets is best known, decision-makers are somewhat constrained in determining degrees due to the constraint on *α*(*n*), *γ*(*n*), and *β*(*n*). Sometimes the total of their membership degrees exceeds 1. In this case, P-FS fails to produce a reasonable result. We will use this circumstance as an example in contradiction of the membership degrees: the choices are, in order, (3/5), (1/5), and (3/5). This makes up for the fact that their sum exceeds 1 and P-FS cannot handle this kind of data. To address these issues, Ashraf [15] presented an innovative structure by establishing spherical fuzzy sets (SFSs), that expand the space for membership levels *α*(*n*), *γ*(*n*), and *β*(*n*) to a somewhat larger extent by satisfying the condition that 0 ≤ *α*^2^(*n*) + *γ*^2^(*n*) + *β*^2^(*n*)≤1. In comparison to past notions, this Ashraf’s structure is significantly more in accordance with human nature, making it one of the most active areas of study today. Ashraf also introduced the aggregation operators [16], dombi aggregation operators [17], t-norms and t-conorms [18], logarithmic aggregation operators [19], emergency support modelling or COVID-19 [20], GRA method [21], TOPSIS method [22] for SFS, etc. It is also playing a very significant role in decision-making, as [23–25].

Making decisions is an essential part of everyday life. Decision-making is the method of selecting the most suitable alternate among a number of alternatives. This last phase of the planning process is essential. How productive you are will depend on the decisions you have made in both your professional and personal lives. If you only have one option open to you, making a choice won’t be difficult. It becomes a challenging process if you are forced to choose from several excellent possibilities. High performance and high-quality outcomes are only feasible in practice if the research community focuses on overcoming theoretical knowledge gaps and practitioners apply the most recent advancements in their applications to tackle real-world issues. As a result, spherical fuzzy *Z̆*-numbers must be introduced to draw on the most recent developments in fuzzy sets, systems, and decision-making, as well as the related significant business applications. The major goal of this research is to develop the foundation for a new model, the spherical fuzzy *Z̆*-number model, which is incredibly adept at expressing ambiguous information. It can be used as a useful tool for making actual uncertain decisions, improving the accuracy of the information used to make decisions, and reflecting their fuzziness, flexibility, and applicability, as shown in Table 1.

**Table 1:** Superiority of SF*Z̆*-Ns over other fuzzy sets.

Sets	*α*(*n*)	*γ*(*n*)	*β*(*n*)	Reliability	Range
Fuzzy Set [1]	✓	×	×	×	0 ≤ *α*(*n*)≤1
*Z̆*-Numbers [3]	✓	×	×	✓	0 ≤ *α*(*Y*, *T*)(*n*) ≤ 1
IFS [10]	✓	×	✓	×	0 ≤ *α*(*n*) + *β*(*n*) ≤ 1
PFS [11]	✓	×	✓	×	0 ≤ *α*^2^(*n*)+ *β*^2^(*n*) ≤ 1
P-FS [13]	✓	✓	✓	×	0 ≤ *α*(*n*) + *γ*(*n*) + *β*(*n*) ≤ 1
SFS [15]	✓	✓	✓	×	0 ≤ *α*^2^(*n*) + *γ*^2^(*n*) + *β*^2^(*n*) ≤ 1
SF*Z̆*-Ns	✓	✓	✓	✓	0 ≤ *α*^2^(*Y*, *T*)(*n*) + *γ*^2^(*Y*, *T*)(*n*) + *β*^2^(*Y*, *T*)(*n*) ≤ 1

It also has several potential applications in the domains of economics, risk analysis, and decision-making. We briefly discussed the procedure for the evaluation of policy impact using SF*Z̆*-Ns. First and foremost, evaluation of policies’ impact is crucial because it lets the government see the outcomes of their efforts (or policies), enables them to be more specific, and identifies places where changes might have an even greater social and economic effect. Therefore, decision making mechanisms or tools must be close to human nature, most reliable, and deprived of one’s preferences. Existing theories are incapable of producing adequate outcomes under these conditions. To overcome this problem, we developed the notion of SF*Z̆*-Ns consisting of 3-D data or information which plays a key role in our ability to make quick and precise judgments.

We will also discuss the powerful technique (TODIM) for ranking alternatives. Many techniques for MADM problems have been offered including the TOPSIS method [26], VIKOR method [27], TODIM method [27], GRA method [28], EDAS method [29], MABAC method [30], MOORA method [31], etc. Many scholars used the TODIM approach for solving MADM problems such as the supplier selection problem [32], robot evaluation [33], robustness analysis framework [34], EDM of bidirectional projection [35], portfolio evaluation [36] and many more. The TODIM approach has the following advantages:

- A strong logic that captures the reasoning behind individual decision-making.
- A scaled value that simultaneously records the best and worst options.
- An easy-to-implement computing technique that can be coded into a spreadsheet.

Therefore, TODIM is chosen as the central development body in this investigation. However, the key flaw of conventional TODIM is its inability to deal with ambiguity and incomplete information while making decisions. Also, it still has a few flaws, like non-discriminatory and counter-intuitive issues. Therefore, spherical fuzzy sets and fuzzy *Z̆*-numbers will be applied with traditional TODIM in order to address this weakness.

The rest of the article is organized as follows: In Section 2, we present the basic preliminaries and related operators. In Section 3, we developed the SF*Z̆*Ns, their fundamental operators, score and accuracy function, and distance formula. In Section 4 we introduced the aggregation operators such as the SF *Z̆*-number weighted averaging operator, SF *Z̆*-number ordered weighted averaging operator, SF *Z̆*-number hybrid averaging operator, SF *Z̆*-number weighted geometric operator, SF *Z̆*-number ordered weighted geometric operator, SF *Z̆*-number hybrid geometric operator. We also developed their theorems, proofs, and properties with proofs. In Section 5, we demonstrate the algorithm for MADM and illustrate the numerical example for decision-making based on SF*Z̆*Ns information to choose the best province that has the greatest impact of policies on the economy. In Section 6, we presented the TODIM approach and numerical examples for understanding and checking the validity of our proposed work. In Section 7 we discussed the relative comparison. In Section 8, we provided the discussion analysis, and we finally concluded our work in Section 9.

## 2 Preliminaries

Few essential definitions and operations that contributed in the creation of the suggested work are introduced in this section.

**Definition 2.1**. [1] Suppose N is the universal set then fuzzy set defined as:

$$\begin{array}{c} \bar{A}=\{\left\langle n,\mu_{\bar{A}}\left(n\right)\right\rangle |n\in N\} \end{array}$$

where $\mu_{\bar{A}}\left(n\right)$ is a membership grade of *n* in $\bar{A}$ and $\mu_{\bar{A}}:N\to \left[0,1\right]$.

**Definition 2.2**. [3] A *Z̆*-number is an ordered pair of fuzzy numbers, represented by by *Z̆* = (*Y*, *W*). The Y component is the membership while W is the reliability of the Y.

**Definition 2.3**. [15] Assuming N is the universal set, the spherical fuzzy set is defined as a non-empty set by the following formula::

$$\begin{array}{c} \tilde{B}=\{\langle n,(\alpha (n),\beta (n),\gamma (n))\rangle |n\in N\}, \end{array}$$

such that *α*(*n*): *N* → [0, 1], *β*(*n*):*N* → [0, 1] and *γ*(*n*):*N* → [0, 1] are the membership, indeterminacy and non-membership grades in a set N with the limitations that 0 ≤ *α*^2^(*n*) + *γ*^2^(*n*) + *β*^2^(*n*) ≤ 1.

**Definition 2.4**. Suppose ${\tilde{B}}_{1}=(\alpha_{1},\beta_{1},\gamma_{1})$ and ${\tilde{B}}_{2}=(\alpha_{2},\beta_{2},\gamma_{2})$ be two SF*Z̆*Ns. Then by the following relations:

(1) ${\overset{`}{B}}_{1}⊇{\tilde{B}}_{2}\Leftrightarrow \alpha_{1}$ ≥ *α*_2_, *β*_1_ ≥ *β*_2_, and *γ*_1_ ≤ *γ*_2_;
(2) ${\overset{`}{B}}_{1}={\overset{`}{B}}_{2}\Leftrightarrow {\overset{`}{B}}_{1}⊇{\overset{`}{B}}_{2}$ and ${\overset{`}{B}}_{2}⊇{\overset{`}{B}}_{1};$
(3) ${\overset{`}{B}}_{1}\cup {\overset{`}{B}}_{2}=(\alpha_{1}\;\vee \alpha_{2},\beta_{1}\wedge \beta_{2},\gamma_{1}\wedge \gamma_{2});$
(4) ${\overset{`}{B}}_{1}\cap {\overset{`}{B}}_{2}=(\alpha_{1}\;\wedge \alpha_{2},\beta_{1}\wedge \beta_{2},\gamma_{1}\vee \gamma_{2});$
(5) ${\left({\overset{`}{B}}_{1}\right)}^{c}={\left(\alpha_{1},\beta_{1},\gamma_{1}\right)}^{c}=(\gamma_{1},\beta_{1},\alpha_{1});$

## 3 Spherical fuzzy *Z̆*-Numbers and its operations

In this section we developed the notion of SF*Z̆*Ns, their fundamental operators and their theorems.

**Definition 3.1**. Suppose N is the universal set then spherical fuzzy *Z̆*-numbers is defined as the subsequent form:

$$\begin{array}{c} S_{Q}=\{\langle n,(\alpha (Y,W)(n),\beta (Y,W)(n),\gamma (Y,W)(n))\rangle |n\in N\}, \end{array}$$

such that *α*(*Y*, *W*)(*n*) = (*α*_*Y*_(*n*), *α*_*W*_(*n*)), *β*(*Y*, *W*)(*n*) = (*β*_*Y*_(*n*), *β*_*W*_(*n*) and *γ*(*Y*, *W*)(*n*) = (*γ*_*Y*_(*n*), *γ*_*W*_(*n*)) such that *α*(*Y*, *W*)(*n*): *N* → [0, 1], *β*(*Y*, *W*)(*n*): *N* → [0, 1] and *γ*(*Y*, *W*)(*n*): *N* → [0, 1] are the order pair of membership, indeterminacy and no-membership grades in a set N and second component W is spherical measures of integrity for Y, along all the conditions

$$\begin{array}{c} 0\leq {\left(\alpha_{Y}\left(n\right)\right)}^{2}+{\left(\beta_{Y}\left(n\right)\right)}^{2}+{\left(\gamma_{Y}\left(n\right)\right)}^{2}\leq 1 \end{array}$$

and

$$\begin{array}{c} 0\leq {\left(\alpha_{W}\left(n\right)\right)}^{2}+{\left(\beta_{W}\left(n\right)\right)}^{2}+{\left(\gamma_{W}\left(n\right)\right)}^{2}\leq 1. \end{array}$$

The element for the standard representation is (*α*(*Y*, *W*)(*n*), *β*(*Y*, *W*)(*n*), *γ*(*Y*, *W*)(*n*)) in *S*_*Q*_ where

(*α*(*Y*, *W*), *β*(*Y*, *W*), *γ*(*Y*, *W*)) = ((*α*_*Y*_, *α*_*W*_), (*β*_*Y*_, *β*_*W*_), (*γ*_*Y*_, *γ*_*W*_)) which is named as SF*Z̆*N.

**Definition 3.2**. Suppose *S*_*Q*1_ = 〈*α*_1_(*Y*, *W*), *β*_1_(*Y*, *W*), *γ*_1_(*Y*, *W*)〉 = 〈(*α*_*Y*1_, *α*_*W*1_)(*β*_*Y*1_, *β*_*W*1_), (*γ*_*Y*1_, *γ*_*W*1_)〉 and *S*_*Q*2_ = 〈*α*_2_(*Y*, *W*), *β*_2_(*Y*, *W*), *γ*_2_(*Y*, *W*)〉 = 〈(*α*_*Y*2_, *α*_*W*2_)(*β*_*Y*2_, *β*_*W*2_), (*γ*_*Y*2_, *γ*_*W*2_)〉 be two SF*Z̆*Ns and λ > 0. Then by the following relations:

(1) *S*_*Q*1_ ⊇ *S*_*Q*2_ ⇔ *α*_*Y*1_ ≥*α*_*Y*2_, *α*_*W*1_ ≥ *α*_*W*2_, *β*_*Y*1_ ≥ *β*_*Y*2_, *β*_*W*1_ ≥ *β*_*W*2_, *γ*_*Y*1_ ≤ *γ*_*Y*2_, *γ*_*W*1_ ≤ *γ*_*W*2_;
(2) *S*_*Q*1_ = *S*_*Q*2_ ⇔ *S*_*Q*1_ ⊇ *S*_*Q*2_ and *S*_*Q*2_ ⊇ *S*_*Q*1_;
(3) *S*_*Q*1_∪*S*_*Q*2_ = 〈(*α*_*Y*1_ ∨*α*_*Y*2_, *α*_*W*1_∨*α*_*W*2_), (*β*_*Y*1_∧*β*_*Y*2_, *β*_*W*1_∧*β*_*W*2_), (*γ*_*Y*1_∧*γ*_*Y*2_, *γ*_*W*1_∧*γ*_*W*2_)〉;
(4) *S*_*Q*1_∩*S*_*Q*2_ = 〈(*α*_*Y*1_ ∧*α*_*Y*2_, *α*_*W*1_∧*α*_*W*2_), (*β*_*Y*1_∧*β*_*Y*2_, *β*_*W*1_∧*β*_*W*2_), (*γ*_*Y*1_∨*γ*_*Y*2_, *γ*_*W*1_∨*γ*_*W*2_)〉;
(5) (*S*_*Q*1_)^*c*^ = 〈*α*_1_(*Y*, *W*), *β*_1_(*Y*, *W*), *γ*_1_(*Y*, *W*)〉^*c*^ = 〈*γ*_1_(*Y*, *W*), *β*_1_(*Y*, *W*), *α*_1_(*Y*, *W*)〉;
(6) $S_{Q1}⊕S_{Q2}=(\begin{array}{c} (\sqrt{\alpha_{Y1}^{2}\;+\alpha_{Y2}^{2}-\alpha_{Y1}^{2}\;\alpha_{Y2}^{2}},\sqrt{\alpha_{W1}^{2}+\alpha_{W2}^{2}-\alpha_{W1}^{2}\alpha_{W2}^{2}}),\\ \left(\beta_{Y1}\beta_{Y2},\beta_{W1}\beta_{W2}\right),\left(\gamma_{Y1}\gamma_{Y2},\gamma_{W1}\gamma_{W2}\right) \end{array});$
(7) $S_{Q1}⊗S_{Q2}=(\begin{array}{c} \left(\alpha_{Y1}\;\alpha_{Y2},\alpha_{W1}\alpha_{W2}\right),\left(\beta_{Y1}\beta_{Y2},\beta_{W1}\beta_{W2}\right),\\ \left(\sqrt{\gamma_{Y1}^{2}+\gamma_{Y2}^{2}-\gamma_{Y1}^{2}\gamma_{Y2}^{2}},\sqrt{\gamma_{W1}^{2}+\gamma_{W2}^{2}-\gamma_{W1}^{2}\gamma_{W2}^{2}}\right) \end{array});$
(8) $\lambda S_{Q1}\;\;\;\;\;\;\;=((\sqrt{1-{\left(1-\alpha_{Y_{1}}^{2}\right)}^{\lambda }},\sqrt{1-{\left(1-\alpha_{W_{1}}^{2}\right)}^{\lambda }}),\left(\beta_{Y_{1}}^{\lambda },\beta_{W_{1}}^{\lambda }\right),\left(\gamma_{Y_{1}}^{\lambda },\gamma_{W_{1}}^{\lambda }\right));$
(9) ${\left(S_{Q1}\right)}^{\lambda }\;\;\;\;=\left(\alpha_{Y1}^{\lambda },\alpha_{W1}^{\lambda }\right)\left(\beta_{Y1}^{\lambda },\beta_{W1}^{\lambda }\right)(\sqrt{1-{\left(1-\gamma_{Y1}^{2}\right)}^{\lambda },}\sqrt{1-{\left(1-\gamma_{W1}^{2}\right)}^{\lambda }}).$

**Definition 3.3**. To compare SF*Z̆*Ns *S*_*Qv*_ = 〈(*α*_*v*_(*Y*, *W*), *β*_*v*_(*Y*, *W*), *γ*_*v*_(*Y*, *W*)〉 = $\langle \left(\alpha_{Y_{v}},\alpha_{W_{v}}\right)\left(\beta_{Y_{v}},\beta_{W_{v}}\right),\left(\gamma_{Y_{v}},\gamma_{W_{v}}\right)\rangle$. We introduce the score function as follows:

$$\begin{array}{c} ℑ\left(S_{Qv}\right)=\frac{2+\left(\alpha_{Y_{v}}\cdot \alpha_{W_{v}}\right)-\left(\beta_{Y_{v}}\cdot \beta_{W_{v}}\right)-\left(\gamma_{Y_{v}}\cdot \gamma_{W_{v}}\right)}{3} \end{array}$$

where, ℑ(*S*_*Qv*_) ∈ [0, 1].

**Definition 3.4**. For any two score values of SF*Z̆*Ns ℑ(*S*_*Qv*_) and ℑ(*S*_*Qv*′_). Then

(1) if ℑ(*S*_*Qv*_) ≥ ℑ(*S*_*Qv*′_) then *S*_*Qv*_ ≥ *S*_*Qv*′_,
(2) if ℑ(*S*_*Qv*_) ≤ ℑ(*S*_*Qv*′_) then *S*_*Qv*_ ≤ *S*_*Qv*′_,
(3) if ℑ(*S*_*Qv*_) = ℑ(*S*_*Qv*′_) then
  calculate the accuracy function:
  $$\begin{array}{c} ℘\left(S_{Qv}\right)=\left(\alpha_{Y_{v}}\cdot \alpha_{W_{v}}\right)-\left(\beta_{Y_{v}}\cdot \beta_{W_{v}}\right),and \end{array}$$
(4) if ℘(*S*_*Qv*_) ≥ ℘(*S*_*Qv*′_) then *S*_*Qv*_ ≥ *S*_*Qv*′_,
(5) if ℘(*S*_*Qv*_) ≤ ℘(*S*_*Qv*′_) then *S*_*Qv*_ ≤ *S*_*Qv*′_,
(6) if ℘(*S*_*Qv*_) = ℘(*S*_*Qv*′_) then *S*_*Qv*_ ∼ *S*_*Qv*′_.

**Example 3.5**. Take two SF*Z̆*Ns as *S*_*Q*1_ = 〈(0.7, 0.8), (0.1, 0.7), (0.3, 0.8)〉 and *S*_*Q*2_ = 〈(0.6, 0.9), (0.3, 0,8), (0.2, 0.7)〉. Their ranking is then listed as follows:

By using the scoring function’s formula, we have

ℑ(*S*_*Q*1_) = {2 + 0.7 × 0.8 − 0.1 × 0.7 − 0.3 × 0.8}/3 = 0.75 and

ℑ(*S*_*Q*2_) = {2 + 0.6 × 0.9 − 0.3 × 0.8 − 0.2 × 0.7}/3 = 0.72.

Since ℑ(*S*_*Q*1_) ≥ ℑ(*S*_*Q*2_) this implies that *S*_*Q*1_ ≥ *S*_*Q*2_.

**Definition 3.6**. Suppose *S*_*Q*1_ = 〈*α*_1_(*Y*, *W*), *β*_1_(*Y*, *W*), *γ*_1_(*Y*, *W*)〉 = 〈(*α*_*Y*1_, *α*_*W*1_)(*β*_*Y*1_, *β*_*W*1_), (*γ*_*Y*1_, *γ*_*W*1_)〉 and *S*_*Q*2_ = 〈*α*_2_(*Y*, *W*), *β*_2_(*Y*, *W*), *γ*_2_(*Y*, *W*)〉 = 〈(*α*_*Y*2_, *α*_*W*2_)(*β*_*Y*2_, *β*_*W*2_), (*γ*_*Y*2_, *γ*_*W*2_)〉 be two SF*Z̆*Ns, then the Euclidean distance between them as follows:

$$\begin{array}{c} d\left(S_{Q1},S_{Q2}\right)=\sqrt{{\left(\left(\alpha_{Y1}\cdot \alpha_{W1}\right)-\left(\alpha_{Y2}\cdot \alpha_{W2}\right)\right)}^{2}+{\left(\left(\beta_{Y1}\cdot \beta_{W1}\right)-\left(\beta_{Y2}\cdot \beta_{W2}\right)\right)}^{2}+{\left(\left(\gamma_{Y1}\cdot \gamma_{W1}\right)-\left(\gamma_{Y2}\cdot \gamma_{W2}\right)\right)}^{2}} \end{array}$$

## 4 Aggregation operators of spherical fuzzy *Z̆*-numbers

In this section we will look at some arithmetic and geometric aggregation operators that use SF *Z̆*-numbers such as the SF *Z̆*-number weighted averaging operator (SF*Z̆*NWA), the SF*Z̆*-number ordered weighted averaging operator (SF*Z̆*NOWA), the SF *Z̆*-number hybrid averaging operator (SF*Z̆*NHA), the SF *Z̆*-number weighted geometric operator (SF*Z̆*NWG), the SF *Z̆*-number ordered weighted geometric operator (SF*Z̆*NOWG), the SF *Z̆*-number hybrid geometric operator (SF*Z̆*NHG).

### 4.1 Spherical fuzzy *Z̆*-number weighted arithmetic operators

**Definition 4.1**. Suppose *S*_*Qv*_ = [(*α*_*v*_(*Y*, *W*), *β*_*v*_(*Y*, *W*), *γ*_*v*_(*Y*, *W*)] = ((*α*_*Yv*_, *α*_*Wv*_)(*β*_*Yv*_, *β*_*Wv*_), (*γ*_*Yv*_, *γ*_*Wv*_)) and *v* = (1, 2, 3…., *g*) be a group of SF*Z̆*Ns and the SF*Z̆*NWA operator such that SF*Z̆*NWA: *SFZ̆N*^*X*^ → *SFZ̆N* is specified as:

$$\begin{array}{c} SF\overset{˘}{Z}NWA\left(S_{Q1},S_{Q2},S_{Q3},...,S_{Qg}\right)=\sum_{v=1}^{g}\lambda_{v}S_{Qv}, \end{array}$$

such that 0 ≤ λ_*v*_ ≤ 1 with $\sum_{v=1}^{g}\lambda_{v}=1$ and λ_*v*_ represents *S*_*Qv*_(*v* = 1, 2, 3, …, *g*) weight vector.

**Theorem 4.2**. *Suppose S*_*Qv*_ = ((*α*_*v*_(*Y*, *W*), *β*_*v*_(*Y*, *W*), *γ*_*v*_(*Y*, *W*)) = ((*α*_*Yv*_, *α*_*Wv*_)(*β*_*Yv*_, *β*_*Wv*_), (*γ*_*Yv*_, *γ*_*Wv*_)) *and v* = (1, 2, 3…, *g*) *be the SFZ̆Ns. Then the aggregated value of the SFZ̆NWA is a SFZ̆N, by using the definition 3.2, we have*:

$$\begin{array}{c} SF\overset{˘}{Z}NWA\left(S_{Q1},S_{Q2},S_{Q3},...,S_{Qg}\right)=\sum_{v=1}^{g}\lambda_{v}S_{Qv}=\\ ((\sqrt{1-\prod_{v=1}^{g}{\left(1-\alpha_{Yv}^{2}\right)}^{\lambda_{v}},}\sqrt{1-\prod_{v=1}^{g}{\left(1-\alpha_{Wv}^{2}\right)}^{\lambda_{v}}}),(\prod_{v=1}^{g}\beta_{Yv}^{\lambda_{v}},\prod_{v=1}^{g}\beta_{Wv}^{\lambda_{v}}),(\prod_{v=1}^{g}\gamma_{Yv}^{\lambda_{v}},\prod_{v=1}^{g}\gamma_{Wv}^{\lambda_{v}})), \end{array}$$

*such that* λ_*v*_ *represents S*_*Qv*_(*v* = 1, 2, 3, …, *g*) *weight vector with* 0 ≤ λ_*v*_ ≤ 1 *and* $\sum_{v=1}^{g}\lambda_{v}=1$.

*Proof*. We will verify the above eq. by using mathematical induction. If *g* = 2, based upon operations (6) and (8) in the Definition 3.2.

We arrive at the following outcome:

$$\begin{array}{c} \lambda_{1}S_{Q1}=(\sqrt{1-{\left(1-\alpha_{Y1}^{2}\right)}^{\lambda_{1}},}\sqrt{1-{\left(1-\alpha_{W1}^{2}\right)}^{\lambda_{1}}}),\left(\beta_{Y1}^{\lambda_{1}},\beta_{W1}^{\lambda_{1}}\right),\left(\gamma_{Y1}^{\lambda_{1}},\gamma_{W1}^{\lambda_{1}}\right) \end{array}$$

$$\begin{array}{c} \lambda_{2}S_{Q2}=(\sqrt{1-{\left(1-\alpha_{Y2}^{2}\right)}^{\lambda_{2}},}\sqrt{1-{\left(1-\alpha_{W2}^{2}\right)}^{\lambda_{2}}}),\left(\beta_{Y2}^{\lambda_{2}},\beta_{W2}^{\lambda_{2}}\right),\left(\gamma_{Y2}^{\lambda_{2}},\gamma_{W2}^{\lambda_{2}}\right) \end{array}$$

$$\begin{array}{c} \begin{array}{cc} SF\overset{˘}{Z}NWA\left(S_{Q1}⊕S_{Q2}\right) & =\sum_{v=1}^{2}\lambda_{v}S_{Qv}=\lambda_{1}S_{Q1}⊕\lambda_{2}S_{Q2}\\ & =((\sqrt{1-{\left(1-\alpha_{Y_{1}}^{2}\right)}^{\lambda_{1}}},\sqrt{1-{\left(1-\alpha_{W_{1}}^{2}\right)}^{\lambda_{1}}}),\left(\beta_{Y_{1}}^{\lambda_{1}},\beta_{W_{1}}^{\lambda_{1}}\right),\left(\gamma_{Y_{1}}^{\lambda_{1}},\gamma_{W_{1}}^{\lambda_{1}}\right))⊕\\ & \;\;\;\;\;((\sqrt{1-{\left(1-\alpha_{Y_{2}}^{2}\right)}^{\lambda_{2}},}\sqrt{1-{\left(1-\alpha_{W_{2}}^{2}\right)}^{\lambda_{2}}}),\left(\beta_{Y_{2}}^{\lambda_{2}},\beta_{W_{2}}^{\lambda_{1}}\right),\left(\gamma_{Y_{2}}^{\lambda_{2}},\gamma_{W_{2}}^{\lambda_{2}}\right))\\ & =(\sqrt{\left(1-{\left(1-\alpha_{Y_{1}}^{2}\right)}^{\lambda_{1}})+(1-{\left(1-\alpha_{Y_{2}}^{2}\right)}^{\lambda_{2}})-(1-{\left(1-\alpha_{Y_{1}}^{2}\right)}^{\lambda_{1}})(1-{\left(1-\alpha_{Y_{2}}^{2}\right)}^{\lambda_{2}}\right)},\\ & \;\;\;\sqrt{\left(1-{\left(1-\alpha_{W_{1}}^{2}\right)}^{\lambda_{1}})+(1-{\left(1-\alpha_{W_{2}}^{2}\right)}^{\lambda_{2}})-(1-{\left(1-\alpha_{W_{1}}^{2}\right)}^{\lambda_{1}})(1-{\left(1-\alpha_{W_{2}}^{2}\right)}^{\lambda_{2}}\right)}),\\ & \;\;\;\;\;\;\;\;\left(\beta_{Y1}^{\lambda_{1}}\beta_{Y2}^{\lambda_{2}},\beta_{W1}^{\lambda_{1}}\beta_{W2}^{\lambda_{2}}\right),\left(\gamma_{Y1}^{\lambda_{1}}\gamma_{Y2}^{\lambda_{2}},\gamma_{W1}^{\lambda_{1}}\gamma_{W2}^{\lambda_{2}}\right)\\ & =(\sqrt{1-{\left(1-\alpha_{Y_{1}}^{2}\right)}^{\lambda_{1}}{\left(1-\alpha_{Y_{2}}^{2}\right)}^{\lambda_{2}})},\sqrt{1-{\left(1-\alpha_{W_{1}}^{2}\right)}^{\lambda_{1}}{\left(1-\alpha_{W_{2}}^{2}\right)}^{\lambda_{2}})}),\\ & \;\;\;\;\;\;\;\;\left(\beta_{Y1}^{\lambda_{1}}\beta_{Y2}^{\lambda_{2}},\beta_{W1}^{\lambda_{1}}\beta_{W2}^{\lambda_{2}}\right),\left(\gamma_{Y1}^{\lambda_{1}}\gamma_{Y2}^{\lambda_{2}},\gamma_{W1}^{\lambda_{1}}\gamma_{W2}^{\lambda_{2}}\right) \end{array} \end{array}$$

this implies that

$$\begin{array}{cc} SF\overset{˘}{Z}NWA\left(S_{Q1}⊕S_{Q2}\right)= & (\sqrt{1-\prod_{v=1}^{2}{\left(1-\alpha_{Yv}^{2}\right)}^{\lambda_{v}},}\sqrt{1-\prod_{v=1}^{2}{\left(1-\alpha_{Wv}^{2}\right)}^{\lambda_{v}}}),\\ & \left(\prod_{v=1}^{2}\beta_{Yv}^{\lambda_{v}},\prod_{v=1}^{2}\beta_{Wv}^{\lambda_{v}}),(\prod_{v=1}^{2}\gamma_{Yv}^{\lambda_{v}},\prod_{v=1}^{2}\gamma_{Wv}^{\lambda_{v}}\right) \end{array}$$

If *g* = *b* then:

$$\begin{array}{c} \text{SF}\overset{˘}{Z}\text{NWA}\;\left(S_{Q1},S_{Q2},S_{Q3},...,S_{Qb}\right)=\sum_{v=1}^{b}\lambda_{v}S_{Qv}=\\ \left((\sqrt{1-\prod_{v=1}^{b}{\left(1-\alpha_{Yv}^{2}\right)}^{\lambda_{v}},}\sqrt{1-\prod_{v=1}^{b}{\left(1-\alpha_{Wv}^{2}\right)}^{\lambda_{v}}}),(\prod_{v=1}^{b}\beta_{Yv}^{\lambda_{v}},\prod_{v=1}^{b},\beta_{Wv}^{\lambda_{v}}),(\prod_{v=1}^{b}\gamma_{Yv}^{\lambda_{v}},\prod_{v=1}^{b}\gamma_{Wv}^{\lambda_{v}})\right) \end{array}$$

If *g* = *b* + 1 then,

$$\begin{array}{c} SF\overset{˘}{Z}NWA\left(S_{Q1},S_{Q2},S_{Q3},...,S_{Qb},S_{Qb+1}\right)=\sum_{v=1}^{b}\lambda_{v}S_{Qv}⊕\lambda_{b+1}S_{Qb+1}\\ =((\sqrt{1-\prod_{v=1}^{b}{\left(1-\alpha_{Yv}^{2}\right)}^{\lambda_{v}},}\sqrt{1-\prod_{v=1}^{b}{\left(1-\alpha_{Wv}^{2}\right)}^{\lambda_{v}}}),(\prod_{v=1}^{b}\beta_{Yv}^{\lambda_{v}},\prod_{v=1}^{b},\beta_{Wv}^{\lambda_{v}}),(\prod_{v=1}^{b}\gamma_{Yv}^{\lambda_{v}},\prod_{v=1}^{b}\gamma_{Wv}^{\lambda_{v}}))⊕\lambda_{b+1}S_{Qb+1}\\ =((\sqrt{1-\prod_{v=1}^{b+1}{\left(1-\alpha_{Yv}^{2}\right)}^{\lambda_{v}},}\sqrt{1-\prod_{v=1}^{b+1}{\left(1-\alpha_{Wv}^{2}\right)}^{\lambda_{v}}}),(\prod_{v=1}^{b+1}\beta_{Yv}^{\lambda_{v}},\prod_{v=1}^{b+1},\beta_{Wv}^{\lambda_{v}}),(\prod_{v=1}^{b+1}\gamma_{Yv}^{\lambda_{v}},\prod_{v=1}^{b+1}\gamma_{Wv}^{\lambda_{v}})). \end{array}$$

This implies that b+1 holds. Hence it is true for all g and it completes the proof.

**Property 4.3**. *Idempotency: Suppose S*_*Qv*_ = (*α*_*v*_(*Y*, *W*), *β*_*v*_(*Y*, *W*), *γ*_*v*_(*Y*, *W*)), *v* ∈ *N be a group of SFZ̆Ns, if all the S*_*Qv*_ *are identical then*

$$\begin{array}{c} SF\overset{˘}{Z}NWA\left(S_{Q1},S_{Q2},S_{Q3},...,S_{Qg}\right)=S_{Q}. \end{array}$$

*Proof*. Suppose all the *S*_*Qv*_ are identical and we know that:

$$\begin{array}{c} SF\overset{˘}{Z}NWA\left(S_{Q1},S_{Q2},S_{Q3},...,S_{Qg}\right)=\sum_{v=1}^{g}\lambda_{v}S_{Qv}\\ =((\sqrt{1-\prod_{v=1}^{g}{\left(1-\alpha_{Yv}^{2}\right)}^{\lambda_{v}}},\sqrt{1-\prod_{v=1}^{g}{\left(1-\alpha_{Wv}^{2}\right)}^{\lambda_{v}}}),(\prod_{v=1}^{g}\beta_{Yv}^{\lambda_{v}},\prod_{v=1}^{g}\beta_{Wv}^{\lambda_{v}}),(\prod_{v=1}^{g}\gamma_{Yv}^{\lambda_{v}},\prod_{v=1}^{g}\gamma_{Wv}^{\lambda_{v}}))\\ =((\sqrt{1-{\left(1-\alpha_{Yv}^{2}\right)}^{\sum_{v=1}^{g}\lambda_{v}},}\sqrt{1-{\left(1-\alpha_{Wv}^{2}\right)}^{\sum_{v=1}^{g}\lambda_{v}}}),(\beta_{Yv}^{\sum_{v=1}^{g}\lambda_{v}},\beta_{Wv}^{\sum_{v=1}^{g}\lambda_{v}}),(\gamma_{Yv}^{\sum_{v=1}^{g}\lambda_{i}},\gamma_{Wv}^{\sum_{v=1}^{g}\lambda_{v}}))\\ =((\sqrt{1-(1-\alpha_{Yv}^{2})},\sqrt{1-(1-\alpha_{Wv}^{2})}),(\beta_{Yv},\beta_{Wv}),(\gamma_{Yv},\gamma_{Wv}))\\ =S_{Q}. \end{array}$$

**Property 4.4**. *Monotonicity: Suppose S*_*Qv*_ = ((*α*_*v*_(*Y*, *W*), *β*_*v*_(*Y*, *W*), *γ*_*v*_(*Y*, *W*)) *and* $S_{Qv^{\prime }}=(\left(\alpha_{v^{\prime }}\left(Y,W\right),\beta_{v^{\prime }}\left(Y,W\right),\gamma_{v^{\prime }}\left(Y,W\right)\right)$ where *v*, *v*′ ∈ *N be a group of SFZ̆Ns, such that S*_*Qv*_ ⊆ *S*_*Qv*′_. *Then*,

$$\begin{array}{c} SF\overset{˘}{Z}NWA\left(S_{Q1},S_{Q2},S_{Q3},...,S_{Qg}\right)\leq SF\overset{˘}{Z}NWA\left(S_{Q1^{\prime }},S_{Q2^{\prime }},S_{Q3^{\prime }},...,S_{Qg^{\prime }}\right). \end{array}$$

*Proof*. As *S*_*Qv*_ ⊆ *S*_*Qv*′_. Thus *α*_*v*_(*Y*, *W*) ≤ *α*_*v*′_(*Y*, *W*), *β*_*v*_(*Y*, *W*) ≤ *β*_*v*′_(*Y*, *W*), and *γ*_*v*_(*Y*, *W*) ≥ *γ*_*v*′_(*Y*, *W*). This implies that

$$\sqrt{1-\prod_{v=1}^{g}{\left(1-\alpha_{Yv}^{2}\right)}^{\lambda_{v}}}\leq \sqrt{1-\prod_{v^{\prime }=1}^{g}{\left(1-\alpha_{Yv^{\prime }}^{2}\right)}^{\lambda_{v^{\prime }}}},$$

$$\sqrt{1-\prod_{v=1}^{g}{\left(1-\alpha_{Wv}^{2}\right)}^{\lambda_{v}}}\leq \sqrt{1-\prod_{v^{\prime }=1}^{g}{\left(1-\alpha_{Wv^{\prime }}^{2}\right)}^{\lambda_{v^{\prime }}}},$$

$$\prod_{v=1}^{g}\beta_{Yv}^{\lambda_{v}}\leq \prod_{v^{\prime }=1}^{g}\beta_{Yv^{\prime }}^{\lambda_{v^{\prime }}},$$

$$\prod_{v=1}^{g}\beta_{Wv}^{\lambda_{v}}\leq \prod_{v^{\prime }=1}^{g}\beta_{Wv^{\prime }}^{\lambda_{v^{\prime }}},,$$

$$\prod_{v=1}^{g}\gamma_{Yv}^{\lambda_{v}}\geq \prod_{v^{\prime }=1}^{g}\gamma_{Yv^{\prime }}^{\lambda_{v^{\prime }}}$$

$$\prod_{v=1}^{g}\gamma_{Wv}^{\lambda_{v}}\geq \prod_{v^{\prime }=1}^{g}\gamma_{Wv^{\prime }}^{\lambda_{v^{\prime }}}.$$

This implies

$$\left((\sqrt{1-\prod_{v=1}^{g}{\left(1-\alpha_{Yv}^{2}\right)}^{\lambda_{v}}},\sqrt{1-\prod_{v=1}^{g}{\left(1-\alpha_{Wv}^{2}\right)}^{\lambda_{v}}}),(\prod_{v=1}^{g}\beta_{Yv}^{\lambda_{v}},\prod_{v=1}^{g}\beta_{Wv}^{\lambda_{v}}),(\prod_{v=1}^{g}\gamma_{Yv}^{\lambda_{v}},\prod_{v=1}^{g}\gamma_{Wv}^{\lambda_{v}})\right)$$

$$\leq ((\sqrt{1-\prod_{v^{\prime }=1}^{g}{\left(1-\alpha_{Yv^{\prime }}^{2}\right)}^{\lambda_{v^{\prime }}}},\sqrt{1-\prod_{v^{\prime }=1}^{g}{\left(1-\alpha_{Wv^{\prime }}^{2}\right)}^{\lambda_{v^{\prime }}}}),(\prod_{v^{\prime }=1}^{g}\beta_{Yv^{\prime }}^{\lambda_{v^{\prime }}},\prod_{v^{\prime }=1}^{g}\beta_{Wv^{\prime }}^{\lambda_{v^{\prime }}}),(\prod_{v^{\prime }=1}^{g}\gamma_{Yv^{\prime }}^{\lambda_{v^{\prime }}},\prod_{v^{\prime }=1}^{g}\gamma_{Wv^{\prime }}^{\lambda_{v^{\prime }}}))$$

Hence

$$\begin{array}{c} SF\overset{˘}{Z}NWA\left(S_{Q1},S_{Q2},S_{Q3},...,S_{Qg}\right)\leq SF\overset{˘}{Z}NWA\left(S_{Q1^{\prime }},S_{Q2^{\prime }},S_{Q3^{\prime }},...,S_{Qg^{\prime }}\right). \end{array}$$

**Property 4.5**. *Boundedness: Suppose S*_*Qv*_ = (*α*_*v*_(*Y*, *W*), *β*_*v*_(*Y*, *W*), *γ*_*v*_(*Y*, *W*)) *for all v* ∈ **N** *be the SFZ̆Ns, such that* $S_{Qı}=\max_{v}S_{Qv}$ *and* $S_{Qȷ}=\min_{v}S_{Qv}$. *Then*,

$$\begin{array}{c} S_{Qȷ}\leq SF\overset{˘}{Z}NWA\left(S_{Q1},S_{Q2},S_{Q3},...,S_{Qg}\right)\leq S_{Qı}. \end{array}$$

*Proof*. The proof is straight forward.

**Definition 4.6**. Suppose *S*_*Q*1_ = (*α*_*v*_(*Y*, *W*), *β*_*v*_(*Y*, *W*), *γ*_*v*_(*Y*, *W*)) = ((*α*_*Yv*_, *α*_*Wv*_)(*β*_*Yv*_, *β*_*Wv*_), (*γ*_*Yv*_, *γ*_*Wv*_)) and *v* = (1, 2, 3…., *g*) be a group of SF*Z̆*Ns and the SF*Z̆*NOWA operator such that SF*Z̆*NOWA: *SFZ̆N*^*X*^ → *SFZ̆N* is specified as:

$$\begin{array}{c} SF\overset{˘}{Z}NOWA\left(S_{Q1},S_{Q2},.....,S_{Qg}\right)=\sum_{v=1}^{g}\lambda_{v}S_{Q_{\delta (v)}} \end{array}$$

with *g* dimensions, such that vth highest weighted value is *S*_*Qv*_ as a result, by the overall order *S*_*Q*1_ ≥ *S*_*Q*2_ ≥ ….≥*S*_*Qg*_ and the weight vector λ_*v*_ = {λ_1_, λ_2_, …., λ_*g*_} with λ_*v*_ ≥ 0 (*v* ∈ **N**) and $\sum_{v=1}^{g}\lambda_{v}=1.$

**Theorem 4.7**. *Suppose S*_*Qv*_ = ((*α*_*v*_(*Y*, *W*), *β*_*v*_(*Y*, *W*), *γ*_*v*_(*Y*, *W*)) = ((*α*_*Yv*_, *α*_*Wv*_)(*β*_*Yv*_, *β*_*Wv*_), (*γ*_*Yv*_, *γ*_*Wv*_)) *and v* = (1, 2, 3…, *g*) *be the SFZ̆Ns. Then aggregated value of the SFZ̆NOWA is a SFZ̆N, by using the definition 3.2, we have*:

$$\begin{array}{c} SF\overset{˘}{Z}NOWA\left(S_{Q1},S_{Q2},.....,S_{Qg}\right)=\sum_{v=1}^{g}\lambda_{v}S_{Q_{\delta (v)}}=\\ ((\sqrt{1-\prod_{v=1}^{2}{\left(1-\alpha_{Y_{\delta (v)}}^{2}\right)}^{\lambda_{v}},}\sqrt{1-\prod_{v=1}^{2}{\left(1-\alpha_{W_{\delta (v)}}^{2}\right)}^{\lambda_{v}}}),(\prod_{v=1}^{2}\beta_{Y_{\delta (v)}}^{\lambda_{v}},\prod_{v=1}^{2}\beta_{W_{\delta (v)}}^{\lambda_{v}}),(\prod_{v=1}^{2}\gamma_{Y_{\delta (v)}}^{\lambda_{v}},\prod_{v=1}^{2}\gamma_{W_{\delta (v)}}^{\lambda_{v}})), \end{array}$$

*with g dimensions, such that S*_*Qv*_ *is the vth biggest value consequently the total order is S*_*Q*1_ ≥ *S*_*Q*2_ ≥ ….≥*S*_*Qg*_ *and the weight vector* λ_*v*_ = {λ_1_, λ_2_, …., λ_*g*_} with λ_*v*_ ≥ 0 (*v* ∈ **N**) *and* $\sum_{v=1}^{g}\lambda_{v}=1.$

*Proof*. The proof is similar to above SF*Z̆*NWA operator. So we omit it.

**Property 4.8**. *Idempotency: Suppose S*_*Qv*_ = (*α*_*v*_(*Y*, *W*), *β*_*v*_(*Y*, *W*), *γ*_*v*_(*Y*, *W*)), *v* ∈ **N** *be a group of SFZ̆Ns, if all the S*_*Qv*_ *are identical then*

$$\begin{array}{c} SF\overset{˘}{Z}NOWA\left(S_{Q1},S_{Q2},S_{Q3},...,S_{Qg}\right)=S_{Q}. \end{array}$$

**Property 4.9**. *Monotonicity: Suppose S*_*Qv*_ = (*α*_*v*_(*Y*, *W*), *β*_*v*_(*Y*, *W*), *γ*_*v*_(*Y*, *W*)) *and* $S_{Qv^{\prime }}=(\left(\alpha_{v^{\prime }}\left(Y,W\right),\beta_{v^{\prime }}\left(Y,W\right),\gamma_{v^{\prime }}\left(Y,W\right)\right)$ *where v*, *v*′ ∈ **N** *be a group of SFZ̆Ns, such that S*_*Qv*_ ⊆ *S*_*Qv*′_. *Then*,

$$\begin{array}{c} SF\overset{˘}{Z}NOWA\left(S_{Q1},S_{Q2},S_{Q3},...,S_{Qg}\right)\leq SF\overset{˘}{Z}NOWA\left(S_{Q1^{\prime }},S_{Q2^{\prime }},S_{Q3^{\prime }},...,S_{Qg^{\prime }}\right). \end{array}$$

**Property 4.10**. *Boundedness: Suppose S*_*Qv*_ = (*α*_*v*_(*Y*, *W*), *β*_*v*_(*Y*, *W*), *γ*_*v*_(*Y*, *W*)) *for all v* ∈ **N** *be a group of SFZ̆Ns, such that* $S_{Qı}=\max_{v}S_{Qv}$ *and* $S_{Qȷ}=\min_{v}S_{Qv}$. *Then*,

$$\begin{array}{c} S_{Qȷ}\leq SF\overset{˘}{Z}NOWA\left(S_{Q1},S_{Q2},S_{Q3},...,S_{Qg}\right)\leq S_{Qı}. \end{array}$$

The weighted SF*Z̆*Ns averaging operator simply takes into account the significance of the aggregated spherical fuzzy sets themselves. The SF*Z̆*NOWA operator solely considers the ranking order of the aggregated spherical fuzzy sets and its position’s importance. We will also define the SF*Z̆*Ns hybrid weighted aggregation operator to address the drawbacks of the two SF*Z̆*Ns aggregation operators discussed before.

**Definition 4.11**. Suppose *S*_*Q*1_ = (*α*_*v*_(*Y*, *W*), *β*_*v*_(*Y*, *W*), *γ*_*v*_(*Y*, *W*)) = ((*α*_*Yv*_, *α*_*Wv*_)(*β*_*Yv*_, *β*_*Wv*_), (*γ*_*Yv*_, *γ*_*Wv*_)) and *v* = (1, 2, 3…., *g*) be a group of SF*Z̆*Ns and the SF*Z̆*NHA operator such that SF*Z̆*NHA: *SFZ̆N*^*X*^ → *SFZ̆N* is specified as:

$$\begin{array}{c} SF\overset{˘}{Z}NHA\left(S_{Q1},S_{Q2},.....,S_{Qg}\right)=\sum_{v=1}^{g}\lambda_{v}S_{Q\delta (v)}^{\prime } \end{array}$$

with *g* dimensions, such that vth biggest weighted value is *S*_*Qδ*(*v*)_ and $S_{Qv}^{\prime }=g\tau_{v}S_{Qv},\left(v\in N\right)$, λ = {λ_1_, λ_2_, …., λ_*g*_} is the weight vectors such that λ_*v*_ ≥ 0 (*v* ∈ **N**) and $\sum_{v=1}^{g}\lambda_{v}=1$. Also *τ*_*v*_ = (*τ*_1_, *τ*_2_, …, *τ*_*g*_) is the associated weight vector such that *τ*_*v*_ ≥ 0 (*v* ∈ **N**) and $\sum_{v=1}^{g}\tau_{v}=1$ and balancing coefficient is *g*.

**Theorem 4.12**. *Suppose S*_*Qv*_ = (*α*_*v*_(*Y*, *W*), *β*_*v*_(*Y*, *W*), *γ*_*v*_(*Y*, *W*)) = ((*α*_*Yv*_, *α*_*Wv*_)(*β*_*Yv*_, *β*_*Wv*_), (*γ*_*Yv*_, *γ*_*Wv*_)) *and v* = (1, 2, 3…, *g*) *be the SFZ̆Ns. Then aggregated value of the SFZ̆NHA is a SFZ̆N, by using the definition 3.2, we have*:

$$\begin{array}{c} SF\overset{˘}{Z}NHA\left(S_{Q1},S_{Q2},.....,S_{Qg}\right)=\sum_{v=1}^{g}\lambda_{v}S_{Q\delta (v)}^{\prime }=\\ (\sqrt{1-\prod_{v=1}^{g}{\left(1-\alpha {{}^{\prime }}_{Y_{\delta (v)}}^{2}\right)}^{\lambda_{v}},}\sqrt{1-\prod_{v=1}^{g}{\left(1-\alpha {{}^{\prime }}_{W_{\delta (v)}}^{2}\right)}^{\lambda_{v}}},(\prod_{v=1}^{g}\beta {{}^{\prime }}_{Y_{\delta (v)}}^{\lambda_{v}},\prod_{v=1}^{g}\beta {{}^{\prime }}_{W_{\delta (v)}}^{\lambda_{v}}),(\prod_{v=1}^{g}\gamma {{}^{\prime }}_{Y_{\delta (v)}}^{\lambda_{v}},\prod_{v=1}^{g}\gamma {{}^{\prime }}_{W_{\delta (v)}}^{\lambda_{v}})), \end{array}$$

*with g dimensions, such that vth biggest weighted value is S*_*Qδ*(*v*)_ *and* $S_{Qv}^{\prime }=g\tau_{v}S_{Qv},\left(v\in N\right)$, *the weight vector* λ = {λ_1_, λ_2_, …., λ_*g*_} *with* λ_*v*_ ≥ 0 (*v* ∈ **N**) *and* $\sum_{v=1}^{g}\lambda_{v}=1$. *Also* *τ*_*v*_ = (*τ*_1_, *τ*_2_, …, *τ*_*g*_) *is the associated weight vector such that τ*_*v*_ ≥ 0 (*v* ∈ **N**) *and* $\sum_{v=1}^{g}\tau_{v}=1$ *and balancing coefficient is g*.

**Property 4.13**. *Idempotency: Suppose S*_*Qv*_ = [(*α*_*v*_(*Y*, *W*), *β*_*v*_(*Y*, *W*), *γ*_*v*_(*Y*, *W*)], *v* ∈ **N** *be a group of SFZ̆Ns, if all the S*_*Qv*_ *are identical then*

$$\begin{array}{c} SF\overset{˘}{Z}NHA\left(S_{Q1},S_{Q2},S_{Q3},...,S_{Qg}\right)=S_{Q}. \end{array}$$

**Property 4.14**. *Monotonicity: Suppose S*_*Qv*_ = (*α*_*v*_(*Y*, *W*), *β*_*v*_(*Y*, *W*), *γ*_*v*_(*Y*, *W*)) *and* $S_{Qv^{\prime }}=(\left(\alpha_{v^{\prime }}\left(Y,W\right),\beta_{v^{\prime }}\left(Y,W\right),\gamma_{v^{\prime }}\left(Y,W\right)\right)$ *where v*, *v*′ ∈ **N** *be a group of SFZ̆Ns, such that S*_*Qv*_ ⊆ *S*_*Qv*′_. *Then*,

$$\begin{array}{c} SF\overset{˘}{Z}NHA\left(S_{Q1},S_{Q2},S_{Q3},...,S_{Qg}\right)\leq SF\overset{˘}{Z}NHA\left(S_{Q1^{\prime }},S_{Q2^{\prime }},S_{Q3^{\prime }},...,S_{Qg^{\prime }}\right). \end{array}$$

**Property 4.15**. *Boundedness: Suppose S*_*Qv*_ = (*α*_*v*_(*Y*, *W*), *β*_*v*_(*Y*, *W*), *γ*_*v*_(*Y*, *W*)) *for all v* ∈ **N** *be a group of SFZ̆Ns, such that* $S_{Qı}=\max_{v}S_{Qv}$ *and* $S_{Qȷ}=\min_{v}S_{Qv}$. *Then*,

$$\begin{array}{c} S_{Qȷ}\leq SF\overset{˘}{Z}NHA\left(S_{Q1},S_{Q2},S_{Q3},...,S_{Qg}\right)\leq S_{Qı}. \end{array}$$

### 4.2 Spherical fuzzy *Z̆*-number weighted geometric operators

**Definition 4.16**. Suppose *S*_*Q*1_ = (*α*_*v*_(*Y*, *W*), *β*_*v*_(*Y*, *W*), *γ*_*v*_(*Y*, *W*)) = ((*α*_*Yv*_, *α*_*Wv*_)(*β*_*Yv*_, *β*_*Wv*_), (*γ*_*Yv*_, *γ*_*Wv*_)) and *v* = (1, 2, 3…., *g*) be the SF*Z̆*Ns and the SF*Z̆*NWG operator such that SF*Z̆*NWG: *SFZ̆N*^*X*^ → *SFZ̆N* is specified as:

$$\begin{array}{c} SF\overset{˘}{Z}NWG\left(S_{Q1},S_{Q2},S_{Q3},...,S_{Qg}\right)=\prod_{v=1}^{g}S_{Qv}^{\lambda_{v}} \end{array}$$

such that λ_*v*_ represents *S*_*Qv*_(*v* = 1, 2, 3, …, *g*) weight vector with 0 ≤ λ_*v*_ ≤ 1 and $\sum_{v=1}^{g}\lambda_{v}=1$.

**Theorem 4.17**. *Suppose S*_*Qv*_ = (*α*_*v*_(*Y*, *W*), *β*_*v*_(*Y*, *W*), *γ*_*v*_(*Y*, *W*)) = ((*α*_*Yv*_, *α*_*Wv*_)(*β*_*Yv*_, *β*_*Wv*_), (*γ*_*Yv*_, *γ*_*Wv*_)) *and v* = (1, 2, 3…, *g*) *be the SFZ̆Ns. Then aggregated value of the SFZ̆NWG is a SFZ̆N, by using the definition 3.2, we have*:

$$\begin{array}{c} SF\overset{˘}{Z}NWG\left(S_{Q1},S_{Q2},S_{Q3},....,S_{Qg}\right)=\prod_{v=1}^{g}S_{Qv}^{\lambda_{v}}=\\ \left((\prod_{v=1}^{g}\alpha_{Yv}^{\lambda_{v}}\prod_{v=1}^{g},\alpha_{Wv}^{\lambda_{v}}),(\prod_{v=1}^{g}\beta_{Yv}^{\lambda_{v}},\prod_{v=1}^{g}\beta_{Wv}^{\lambda_{v}}),(\sqrt{1-\prod_{v=1}^{g}{\left(1-\gamma_{Yv}^{2}\right)}^{\lambda_{v}},}\sqrt{1-\prod_{v=1}^{g}{\left(1-\gamma_{Wv}^{2}\right)}^{\lambda_{v}}})\right) \end{array}$$

*such that* λ_*v*_ *represents S*_*Qv*_(*v* = 1, 2, 3, …, *g*) *weight vector with* 0 ≤ λ_*v*_ ≤ 1 *and* $\sum_{v=1}^{g}\lambda_{v}=1$.

*Proof*. We will verify the above eq. by using mathematical induction. If *g* = 2, according to operations in definition 3.2

We arrive at the following outcome:

$$\begin{array}{c} S_{Q1}^{\lambda 1}=(\left(\alpha_{Y_{1}}^{\lambda_{1}},\alpha_{W_{1}}^{\lambda_{1}}\right),\left(\beta_{Y_{1}}^{\lambda_{1}},\beta_{W_{1}}^{\lambda_{1}}\right),(\sqrt{1-{\left(1-\gamma_{Y_{1}}^{2}\right)}^{\lambda_{1}}},\sqrt{1-{\left(1-\gamma_{W_{1}}^{2}\right)}^{\lambda_{1}}})) \end{array}$$

$$\begin{array}{c} S_{Q2}^{\lambda 2}=(\left(\alpha_{Y_{2}}^{\lambda_{2}},\alpha_{W_{2}}^{\lambda_{2}}\right),\left(\beta_{Y_{2}}^{\lambda_{2}},\beta_{W_{2}}^{\lambda_{1}}\right),(\sqrt{1-{\left(1-\gamma_{Y_{2}}^{2}\right)}^{\lambda_{2}},}\sqrt{1-{\left(1-\gamma_{W_{2}}^{2}\right)}^{\lambda_{2}}})) \end{array}$$

$$\begin{array}{c} \begin{array}{cc} SF\overset{˘}{Z}NWG\left(S_{Q1}⊗S_{Q2}\right) & =\prod_{v=1}^{2}S_{Qv}^{\lambda_{v}}=S_{Q1}^{\lambda_{1}}⊗S_{Q2}^{\lambda_{2}}\\ & =(\left(\alpha_{Y_{1}}^{\lambda_{1}},\alpha_{W_{1}}^{\lambda_{1}}\right),\left(\beta_{Y_{1}}^{\lambda_{1}},\beta_{W_{1}}^{\lambda_{1}}\right),(\sqrt{1-{\left(1-\gamma_{Y_{1}}^{2}\right)}^{\lambda_{1}}},\sqrt{1-{\left(1-\gamma_{W_{1}}^{2}\right)}^{\lambda_{1}}}))⊗\\ & \;\;\;\;\;(\left(\alpha_{Y_{2}}^{\lambda_{2}},\alpha_{W_{2}}^{\lambda_{2}}\right),\left(\beta_{Y_{2}}^{\lambda_{2}},\beta_{W_{2}}^{\lambda_{1}}\right),(\sqrt{1-{\left(1-\gamma_{Y_{2}}^{2}\right)}^{\lambda_{2}},}\sqrt{1-{\left(1-\gamma_{W_{2}}^{2}\right)}^{\lambda_{2}}}))\\ & =\left(\alpha_{Y1}^{\lambda_{1}}\alpha_{Y2}^{\lambda_{2}},\alpha_{W1}^{\lambda_{1}}\alpha_{W2}^{\lambda_{2}}\right),\left(\beta_{Y1}^{\lambda_{1}}\beta_{Y2}^{\lambda_{2}},\beta_{W1}^{\lambda_{1}}\beta_{W2}^{\lambda_{2}}\right),\\ & \;\;\;\;(\sqrt{\left(1-{\left(1-\gamma_{Y_{1}}^{2}\right)}^{\lambda_{1}})+(1-{\left(1-\gamma_{Y_{2}}^{2}\right)}^{\lambda_{2}})-(1-{\left(1-\gamma_{Y_{1}}^{2}\right)}^{\lambda_{1}})(1-{\left(1-\gamma_{Y_{2}}^{2}\right)}^{\lambda_{2}}\right)},\\ & \;\;\;\;\sqrt{\left(1-{\left(1-\gamma_{W_{1}}^{2}\right)}^{\lambda_{1}})+(1-{\left(1-\gamma_{W_{2}}^{2}\right)}^{\lambda_{2}})-(1-{\left(1-\gamma_{W_{1}}^{2}\right)}^{\lambda_{1}})(1-{\left(1-\gamma_{W_{2}}^{2}\right)}^{\lambda_{2}}\right)})\\ & =\left(\alpha_{Y1}^{\lambda_{1}}\alpha_{Y2}^{\lambda_{2}},\alpha_{W1}^{\lambda_{1}}\alpha_{W2}^{\lambda_{2}}\right),\left(\beta_{Y1}^{\lambda_{1}}\beta_{Y2}^{\lambda_{2}},\beta_{W1}^{\lambda_{1}}\beta_{W2}^{\lambda_{2}}\right),(\sqrt{1-{\left(1-\gamma_{Y_{1}}^{2}\right)}^{\lambda_{1}}{\left(1-\gamma_{Y_{2}}^{2}\right)}^{\lambda_{2}})},\\ & \;\;\;\;\;\;\sqrt{1-{\left(1-\gamma_{W_{1}}^{2}\right)}^{\lambda_{1}}{\left(1-\gamma_{W_{2}}^{2}\right)}^{\lambda_{2}})}) \end{array} \end{array}$$

this implies that

$$\begin{array}{c} \begin{array}{cc} SF\overset{˘}{Z}NWG\left(S_{Q1}⊕S_{Q2}\right)=( & \prod_{v=1}^{2}\alpha_{Yv}^{\lambda_{v}},\prod_{v=1}^{2}\alpha_{Wv}^{\lambda_{v}}),(\prod_{v=1}^{2}\beta_{Yv}^{\lambda_{v}},\prod_{v=1}^{2}\beta_{Wv}^{\lambda_{v}}),\\ & \left(\sqrt{1-\prod_{v=1}^{2}{\left(1-\gamma_{Yv}^{2}\right)}^{\lambda_{v}},}\sqrt{1-\prod_{v=1}^{2}{\left(1-\gamma_{Wv}^{2}\right)}^{\lambda_{v}}}\right) \end{array} \end{array}$$

If *x* = *m* then:

$$\begin{array}{c} \text{SF}\overset{˘}{Z}\text{NWG}\left(S_{Q1},S_{Q2},S_{Q3},...,S_{Qb}\right)=\prod_{v=1}^{b}S_{Qv}^{\lambda_{v}}=\\ \left((\prod_{v=1}^{b}\alpha_{Yv}^{\lambda_{v}},\prod_{v=1}^{b}\alpha_{Wv}^{\lambda_{v}}),(\prod_{v=1}^{b}\beta_{Yv}^{\lambda_{v}},\prod_{v=1}^{b}\beta_{Wv}^{\lambda_{v}}),(\sqrt{1-\prod_{v=1}^{b}{\left(1-\gamma_{Yv}^{b}\right)}^{\lambda_{v}},}\sqrt{1-\prod_{v=1}^{b}{\left(1-\gamma_{Wv}^{b}\right)}^{\lambda_{v}}})\right) \end{array}$$

If *g* = *b* + 1 then,

$$\begin{array}{c} SF\overset{˘}{Z}NWG\left(S_{Q1},S_{Q2},S_{Q3},...,S_{Qb},S_{Qb+1}\right)=\prod_{v=1}^{b}S_{Qv}^{\lambda_{v}}⊕S_{Qb+1}^{\lambda_{b+1}}\\ =((\prod_{v=1}^{b}\alpha_{Yv}^{\lambda_{v}},\prod_{v=1}^{b}\alpha_{Wv}^{\lambda_{v}}),(\prod_{v=1}^{b}\beta_{Yv}^{\lambda_{v}},\prod_{v=1}^{b}\beta_{Wv}^{\lambda_{v}}),(\sqrt{1-\prod_{v=1}^{b}{\left(1-\gamma_{Yv}^{b}\right)}^{\lambda_{v}},}\sqrt{1-\prod_{v=1}^{b}{\left(1-\gamma_{Wv}^{b}\right)}^{\lambda_{v}}}))⊗S_{Qb+1}^{\lambda_{b+1}}\\ =((\prod_{v=1}^{b+1}\alpha_{Yv}^{\lambda_{v}},\prod_{v=1}^{b+1}\alpha_{Wv}^{\lambda_{v}}),(\prod_{v=1}^{b+1}\beta_{Yv}^{\lambda_{v}},\prod_{v=1}^{b+1}\beta_{Wv}^{\lambda_{v}}),(\sqrt{1-\prod_{v=1}^{b+1}{\left(1-\gamma_{Yv}^{b+1}\right)}^{\lambda_{i}},}\sqrt{1-\prod_{v=1}^{b+1}{\left(1-\gamma_{Wv}^{b+1}\right)}^{\lambda_{v}}})). \end{array}$$

This implies that b+1 holds. Hence it is true for all g and it completes the proof.

**Property 4.18**. *Idempotency: Suppose S*_*Qv*_ = (*α*_*v*_(*Y*, *W*), *β*_*v*_(*Y*, *W*), *γ*_*v*_(*Y*, *W*)), *v* ∈ **N** *be a group of SFZ̆Ns, if all the S*_*Qv*_ *are identical then*

$$\begin{array}{c} SF\overset{˘}{Z}NWG\left(S_{Q1},S_{Q2},S_{Q3},...,S_{Qg}\right)=S_{Q}. \end{array}$$

*Proof*. Suppose all the *S*_*Qv*_ are identical and we know that:

$$\begin{array}{c} SF\overset{˘}{Z}NWG\left(S_{Q1},S_{Q2},S_{Q3},...,S_{Qg}\right)=\prod_{v=1}^{g}S_{Qv}^{\lambda_{v}}\\ =((\prod_{v=1}^{b}\alpha_{Yv}^{\lambda_{v}},\prod_{v=1}^{b}\alpha_{Wv}^{\lambda_{v}}),(\prod_{v=1}^{b}\beta_{Yv}^{\lambda_{v}},\prod_{v=1}^{b}\beta_{Wv}^{\lambda_{v}}),(\sqrt{1-\prod_{v=1}^{b}{\left(1-\gamma_{Yv}^{b}\right)}^{\lambda_{v}},}\sqrt{1-\prod_{v=1}^{b}{\left(1-\gamma_{Wv}^{b}\right)}^{\lambda_{v}}}))\\ =((\alpha_{Yv}^{\sum_{v=1}^{b}\lambda_{v}},\alpha_{Wv}^{\sum_{v=1}^{b}\lambda_{v}}),(\beta_{Yv}^{\sum_{v=1}^{b}\lambda_{v}},\beta_{Wv}^{\sum_{v=1}^{b}\lambda_{v}}),(\sqrt{1-{\left(1-\gamma_{Yv}^{b}\right)}^{\sum_{v=1}^{b}\lambda_{v}}},\sqrt{1-{\left(1-\gamma_{Wv}^{b}\right)}^{\sum_{v=1}^{b}\lambda_{v}}}))\\ =((\alpha_{Yv},\alpha_{Wv}),(\beta_{Yv},\beta_{Wv}),(\sqrt{1-(1-\gamma_{Yv}^{b})},\sqrt{1-(1-\gamma_{Wv}^{b})}))\\ =S_{Q}. \end{array}$$

**Property 4.19**. *Monotonicity: Suppose S*_*Qv*_ = (*α*_*v*_(*Y*, *W*), *β*_*v*_(*Y*, *W*), *γ*_*v*_(*Y*, *W*)) and $S_{Qv^{\prime }}=\left(\alpha_{v^{\prime }}\left(Y,W\right),\beta_{v^{\prime }}\left(Y,W\right),\gamma_{v^{\prime }}\left(Y,W\right)\right)$ *where v*, *v*′ ∈ **N** *be a group of SFZ̆Ns, such that S*_*Qv*_ ⊆ *S*_*Qv*′_. *Then*,

$$\begin{array}{c} SF\overset{˘}{Z}NWG\left(S_{Q1},S_{Q2},S_{Q3},...,S_{Qg}\right)\leq SF\overset{˘}{Z}NWG\left(S_{Q1^{\prime }},S_{Q2^{\prime }},S_{Q3^{\prime }},...,S_{Qg^{\prime }}\right). \end{array}$$

*Proof*. As *S*_*Qv*_ ⊆ *S*_*Qv*′_. Thus *α*_*v*_(*Y*, *W*) ≤ *α*_*v*′_(*Y*, *W*), *β*_*v*_(*Y*, *W*) ≤ *β*_*v*′_(*Y*, *W*), and *γ*_*v*_(*Y*, *W*) ≥ *γ*_*v*′_(*Y*, *W*). This implies that

$$\prod_{v=1}^{g}\alpha_{Yv}^{\lambda_{v}}\geq \prod_{v^{\prime }=1}^{g}\alpha_{Yv^{\prime }}^{\lambda_{v^{\prime }}}$$

$$\prod_{v=1}^{g}\alpha_{Wv}^{\lambda_{v}}\geq \prod_{v^{\prime }=1}^{g}\alpha_{Wv^{\prime }}^{\lambda_{v^{\prime }}},$$

$$\prod_{v=1}^{g}\beta_{Yv}^{\lambda_{v}}\leq \prod_{v^{\prime }=1}^{g}\beta_{Yv^{\prime }}^{\lambda_{v^{\prime }}},$$

$$\prod_{v=1}^{g}\beta_{Wv}^{\lambda_{v}}\leq \prod_{v^{\prime }=1}^{g}\beta_{Wv^{\prime }}^{\lambda_{v^{\prime }}},,$$

$$\sqrt{1-\prod_{v=1}^{g}{\left(1-\gamma_{Yv}^{2}\right)}^{\lambda_{v}}}\leq \sqrt{1-\prod_{v^{\prime }=1}^{g}{\left(1-\gamma_{Yv^{\prime }}^{2}\right)}^{\lambda_{v^{\prime }}}},$$

$$\sqrt{1-\prod_{v=1}^{g}{\left(1-\gamma_{Wv}^{2}\right)}^{\lambda_{v}}}\leq \sqrt{1-\prod_{v^{\prime }=1}^{g}{\left(1-\gamma_{Wv^{\prime }}^{2}\right)}^{\lambda_{v^{\prime }}}}.$$

This implies

$$\left((\prod_{v=1}^{g}\alpha_{Yv}^{\lambda_{v}}\prod_{v=1}^{g},\alpha_{Wv}^{\lambda_{v}}),(\prod_{v=1}^{g}\beta_{Yv}^{\lambda_{v}},\prod_{v=1}^{g}\beta_{Wv}^{\lambda_{v}}),(\sqrt{1-\prod_{v=1}^{g}{\left(1-\gamma_{Yv}^{2}\right)}^{\lambda_{v}},}\sqrt{1-\prod_{v=1}^{g}{\left(1-\gamma_{Wv}^{2}\right)}^{\lambda_{v}}})\right)$$

$$\leq ((\prod_{v^{\prime }=1}^{g}\alpha_{Yv^{\prime }}^{\lambda_{v^{\prime }}}\prod_{v^{\prime }=1}^{g},\alpha_{Wv^{\prime }}^{\lambda_{v^{\prime }}}),(\prod_{v^{\prime }=1}^{g}\beta_{Yv^{\prime }}^{\lambda_{v^{\prime }}},\prod_{v^{\prime }=1}^{g}\beta_{Wv^{\prime }}^{\lambda_{v^{\prime }}}),(\sqrt{1-\prod_{v^{\prime }=1}^{g}{\left(1-\gamma_{Yv^{\prime }}^{2}\right)}^{\lambda_{v^{\prime }}},}\sqrt{1-\prod_{v^{\prime }=1}^{g}{\left(1-\gamma_{Wv^{\prime }}^{2}\right)}^{\lambda_{v^{\prime }}}}))$$

Hence

$$\begin{array}{c} SF\overset{˘}{Z}NWG\left(S_{Q1},S_{Q2},S_{Q3},...,S_{Qg}\right)\leq SF\overset{˘}{Z}NWG\left(S_{Q1^{\prime }},S_{Q2^{\prime }},S_{Q3^{\prime }},...,S_{Qg^{\prime }}\right). \end{array}$$

**Property 4.20**. *Boundedness: Suppose S*_*Qv*_ = (*α*_*v*_(*Y*, *W*), *β*_*v*_(*Y*, *W*), *γ*_*v*_(*Y*, *W*)) *for all v* ∈ **N** *be a group of SFZ̆Ns, such that* $S_{Qı}=\max_{v}S_{Qv}$ *and* $S_{Qȷ}=\min_{v}S_{Qv}$. *Then*,

$$\begin{array}{c} S_{Qȷ}\leq SF\overset{˘}{Z}NWG\left(S_{Q1},S_{Q2},S_{Q3},...,S_{Qg}\right)\leq S_{Qı}. \end{array}$$

*Proof*. The proof is straight forward.

**Definition 4.21**. Suppose *S*_*Q*1_ = (*α*_*v*_(*Y*, *W*), *β*_*v*_(*Y*, *W*), *γ*_*v*_(*Y*, *W*)) = ((*α*_*Yv*_, *α*_*Wv*_)(*β*_*Yv*_, *β*_*Wv*_), (*γ*_*Yv*_, *γ*_*Wv*_)) and *v* = (1, 2, 3…., *g*) be a group of SF*Z̆*Ns and the SF*Z̆*NOWG operator such that SF*Z̆*NOWG: *SFZ̆N*^*X*^ → *SFZ̆N* is specified as:

$$\begin{array}{c} SF\overset{˘}{Z}NOWG\left(S_{Q1},S_{Q2},.....,S_{Qg}\right)=\prod_{v=1}^{g}S_{Q\delta (v)}^{\lambda_{v}} \end{array}$$

with *g* dimensions, such that vth highest weighted value is *S*_*Qv*_ as a result, by the overall order *S*_*Q*1_ ≥ *S*_*Q*2_ ≥ ….≥*S*_*Qg*_ and λ_*v*_ = {λ_1_, λ_2_, …., λ_*g*_} is the weight vectors such that λ_*v*_ ≥ 0 (*v* ∈ **N**) and $\sum_{v=1}^{g}\lambda_{v}=1.$

**Theorem 4.22**. *Suppose S*_*Qv*_ = (*α*_*v*_(*Y*, *W*), *β*_*v*_(*Y*, *W*), *γ*_*v*_(*Y*, *W*)) = ((*α*_*Yv*_, *α*_*Wv*_)(*β*_*Yv*_, *β*_*Wv*_), (*γ*_*Yv*_, *γ*_*Wv*_)) *and v* = (1, 2, 3…, *g*) *be the SFZ̆Ns. Then aggregated value of the SFZ̆NOWG is a SFZ̆N, by using the definition 3.2, we have*:

$$\begin{array}{c} SF\overset{˘}{Z}NOWG\left(S_{Q1},S_{Q2},.....,S_{Qg}\right)=\prod_{v=1}^{g}S_{Q\delta (v)}^{\lambda_{v}}=\\ ((\prod_{v=1}^{g}\alpha_{Y_{\delta (v)}}^{\lambda_{v}}\prod_{v=1}^{g},\alpha_{W_{\delta (v)}}^{\lambda_{v}}),(\prod_{v=1}^{g}\beta_{Y_{\delta (v)}}^{\lambda_{v}},\prod_{v=1}^{g}\beta_{W_{\delta (v)}}^{\lambda_{v}}),(\sqrt{1-\prod_{v=1}^{g}{\left(1-\gamma_{Y_{\delta (v)}}^{2}\right)}^{\lambda_{v}},}\sqrt{1-\prod_{v=1}^{g}{\left(1-\gamma_{W_{\delta (v)}}^{2}\right)}^{\lambda_{v}}})), \end{array}$$

*with g dimensions, such that S*_*Qv*_ *is the vth biggest value consequently the total order is S*_*Q*1_ ≥ *S*_*Q*2_ ≥ ….≥*S*_*Qg*_ *and weight vector* λ_*v*_ = {λ_1_, λ_2_, …., λ_*g*_} *with* λ_*v*_ ≥ 0 (*v* ∈ **N**) *and* $\sum_{v=1}^{g}\lambda_{v}=1.$

*Proof*. The proof is similar to above SF*Z̆*NWG operator. So we omit it.

**Property 4.23**. *Idempotency: Suppose S*_*Qv*_ = (*α*_*v*_(*Y*, *W*), *β*_*v*_(*Y*, *W*), *γ*_*v*_(*Y*, *W*)), *v* ∈ **N** *be a group of SFZ̆Ns, if all the S*_*Qv*_ *are identical then*

$$\begin{array}{c} SF\overset{˘}{Z}NOWG\left(S_{Q1},S_{Q2},S_{Q3},...,S_{Qx}\right)=S_{Q}. \end{array}$$

**Property 4.24**. *Monotonicity: Suppose S*_*Qv*_ = (*α*_*v*_(*Y*, *W*), *β*_*v*_(*Y*, *W*), *γ*_*v*_(*Y*, *W*)) *and* $S_{Qv^{\prime }}=(\left(\alpha_{v^{\prime }}\left(Y,W\right),\beta_{iv}\left(Y,W\right),\gamma_{v^{\prime }}\left(Y,W\right)\right)$ *where v*, *v*′ ∈ **N** *be a group of SFZ̆Ns, such that S*_*Qv*_ ⊆ *S*_*Qv*′_. *Then*,

$$\begin{array}{c} SF\overset{˘}{Z}NOWG\left(S_{Q1},S_{Q2},S_{Q3},...,S_{Qg}\right)\leq SF\overset{˘}{Z}NOWG\left(S_{Q1^{\prime }},S_{Q2^{\prime }},S_{Q3^{\prime }},...,S_{Qg^{\prime }}\right). \end{array}$$

**Property 4.25**. *Boundedness: Suppose S*_*Qv*_ = (*α*_*v*_(*Y*, *W*), *β*_*v*_(*Y*, *W*), *γ*_*v*_(*Y*, *W*)) *for all v* ∈ **N** *be a group of SFZ̆Ns, such that* $S_{Qı}=\max_{v}S_{Qv}$ *and* $S_{Qȷ}=\min_{v}S_{Qv}$. *Then*,

$$\begin{array}{c} S_{Qȷ}\leq SF\overset{˘}{Z}NOWG\left(S_{Q1},S_{Q2},S_{Q3},...,S_{Qg}\right)\leq S_{Qı}. \end{array}$$

The weighted SF*Z̆*Ns averaging operator simply takes into account the significance of the aggregated spherical fuzzy sets themselves. The SF*Z̆*NOWG operator solely considers the ranking order of the aggregated spherical fuzzy sets and its position’s importance. We will also define the SF*Z̆*Ns hybrid weighted aggregation operator to address the drawbacks of the two SF*Z̆*Ns aggregation operators discussed before.

**Definition 4.26**. Suppose *S*_*Q*1_ = (*α*_*v*_(*Y*, *W*), *β*_*v*_(*Y*, *W*), *γ*_*v*_(*Y*, *W*)) = ((*α*_*Yv*_, *α*_*Wv*_)(*β*_*Yv*_, *β*_*Wv*_), (*γ*_*Yv*_, *γ*_*Wv*_)) and *v* = (1, 2, 3…., *g*) be a group of SF*Z̆*Ns and the SF*Z̆*NHG operator such that SF*Z̆*NHG: *SFZ̆N*^*X*^ → *SFZ̆N* is specified as:

$$\begin{array}{c} SF\overset{˘}{Z}NHG\left(S_{Q1},S_{Q2},.....,S_{Qg}\right)=\prod_{v=1}^{g}{\left(S_{Q\delta (v)}^{\prime }\right)}^{\lambda_{v}} \end{array}$$

with *g* dimensions, such that vth biggest weighted value is *S*_*Qδ*(*v*)_ and $S_{Qv}^{\prime }=S_{Qv}^{g\tau_{v}},\left(v\in N\right)$, λ = {λ_1_, λ_2_, …., λ_*g*_} is the weight vectors such that λ_*v*_ ≥ 0 (*v* ∈ **N**) and $\sum_{v=1}^{g}\lambda_{v}=1$. Also *τ*_*v*_ = (*τ*_1_, *τ*_2_, …, *τ*_*g*_) is the associated weight vector such that *τ*_*v*_ ≥ 0 (*v* ∈ **N**) and $\sum_{v=1}^{g}\tau_{v}=1$ and balancing coefficient is *g*.

**Theorem 4.27**. *Suppose S*_*Qv*_ = (*α*_*v*_(*Y*, *W*), *β*_*v*_(*Y*, *W*), *γ*_*v*_(*Y*, *W*)) = ((*α*_*Yv*_, *α*_*Wv*_)(*β*_*Yv*_, *β*_*Wv*_), (*γ*_*Yv*_, *γ*_*Wv*_)) *and v* = (1, 2, 3…, *g*) *be a group of SFZ̆Ns. Then the collected value of the SFZ̆NHG is a SFZ̆N, obtained by using the definition 3.2, we have*:

$$\begin{array}{c} SF\overset{˘}{Z}NHG\left(S_{Q1},S_{Q2},.....,S_{Qg}\right)=\prod_{v=1}^{g}{\left(S_{Q\delta (v)}^{\prime }\right)}^{\lambda_{v}}=\\ ((\prod_{v=1}^{g}\alpha {{}^{\prime }}_{Y_{\delta (v)}}^{\lambda_{v}}\prod_{v=1}^{g},\alpha {{}^{\prime }}_{W_{\delta (v)}}^{\lambda_{v}}),(\prod_{v=1}^{g}\beta {{}^{\prime }}_{Y_{\delta (v)}}^{\lambda_{v}},\prod_{v=1}^{g}\beta {{}^{\prime }}_{W_{\delta (v)}}^{\lambda_{v}}),(\sqrt{1-\prod_{v=1}^{g}{\left(1-\gamma {{}^{\prime }}_{Y_{\delta (v)}}^{2}\right)}^{\lambda_{v}},}\sqrt{1-\prod_{v=1}^{g}{\left(1-\gamma {{}^{\prime }}_{W_{\delta (v)}}^{2}\right)}^{\lambda_{v}}})), \end{array}$$

*with g dimensions, such that vth biggest weighted value is S*_*Qδ*(*v*)_ *and* $S_{Qv}^{\prime }=S_{Qv}^{g\tau_{v}},\left(v\in N\right)$, λ = {λ_1_, λ_2_, …., λ_*g*_} *is the weight vectors such that* λ_*v*_ ≥ 0 (*v* ∈ **N**) *and* $\sum_{v=1}^{g}\lambda_{v}=1$. *Also τ*_*v*_ = (*τ*_1_, *τ*_2_, …, *τ*_*g*_) *is the associated weight vector such that τ*_*v*_ ≥ 0 (*v* ∈ **N**) *and* $\sum_{v=1}^{g}\tau_{v}=1$ *and balancing coefficient is g*.

**Property 4.28**. *Idempotency: Suppose S*_*Qv*_ = (*α*_*v*_(*Y*, *W*), *β*_*v*_(*Y*, *W*), *γ*_*v*_(*Y*, *W*)), *v* ∈ **N** *be a group of SFZ̆Ns, if all the S*_*Qv*_ *are identical then*

$$\begin{array}{c} SF\overset{˘}{Z}NHG\left(S_{Q1},S_{Q2},S_{Q3},...,S_{Qx}\right)=S_{Q}. \end{array}$$

**Property 4.29**. *Monotonicity: Suppose S*_*Qv*_ = (*α*_*v*_(*Y*, *W*), *β*_*v*_(*Y*, *W*), *γ*_*v*_(*Y*, *W*)) *and* $S_{Qv^{\prime }}=\left(\alpha_{v^{\prime }}\left(Y,W\right),\beta_{v^{\prime }}\left(Y,W\right),\gamma_{v^{\prime }}\left(Y,W\right)\right)$ *where v*, *v*′ ∈ **N** *be a group of SFZ̆Ns, such that S*_*Qv*_ ⊆ *S*_*Qv*′_. *Then*,

$$\begin{array}{c} SF\overset{˘}{Z}NHG\left(S_{Q1},S_{Q2},S_{Q3},...,S_{Qg}\right)\leq SF\overset{˘}{Z}NHG\left(S_{Q1^{\prime }},S_{Q2^{\prime }},S_{Q3^{\prime }},...,S_{Qg^{\prime }}\right). \end{array}$$

**Property 4.30**. *Boundedness: Suppose S*_*Qv*_ = (*α*_*v*_(*Y*, *W*), *β*_*v*_(*Y*, *W*), *γ*_*v*_(*Y*, *W*)) *for all v* ∈ **N** *be a group of SFZ̆Ns, such that* $S_{Qı}=\max_{v}S_{Qv}$ *and* $S_{Qȷ}=\min_{v}S_{Qv}$. *Then*,

$$\begin{array}{c} S_{Qȷ}\leq SF\overset{˘}{Z}NHG\left(S_{Q1},S_{Q2},S_{Q3},...,S_{Qg}\right)\leq S_{Qı}. \end{array}$$

## 5 An approach to multiple criteria decision making

In this section, an algorithm was created to solve the MCDM problem utilizing the suggested average and geometric aggregation operators, along with a MCDM example.

Suppose the collection of alternatives *P* = {*p*_1_, *p*_2_, …, *p*_*m*_} and the collection of attributes *W* = {*w*_1_, *w*_2_, …, *w*_*g*_} with the weight vectors λ = {λ_1_, λ_2_, …, λ_*g*_}. The weight vector requirement is that weights must belong to a closed unit interval and that their sum must be equal to 1. Then, based on the suggested aggregation operators, we summarized the subsequent steps to determine the best solution among the feasible ones.

**Algorithm 5.1**

***Step 1***: *Consider universal set, weight vectors and attribute’s set as an input then construct the SFZ̆Ns decision matrix as follows S*_*Q*_ = [*S*_*Qjv*_]_*m*×*g*_ *after collecting expert evaluation information regarding each alternative’s attributes*.

***Step 2***: *There are two different types of criteria used widely, one of which is referred to as a positive criterion and the other as a negative criterion. We must change the negative criteria into positive criteria by taking the complement for the negative criterion*.

***Step 3***: *To integrate the attributes for each alternative, use SFZ̆Ns arithmetic and geometric aggregation operators which are discussed above*.

***Step 4***: *Compute the score values by using definition 3.3, 3.4 for each alternative*.

***Step 5***: *Rank the all alternatives in descending order and choose the best one*.

**Fig 1:**
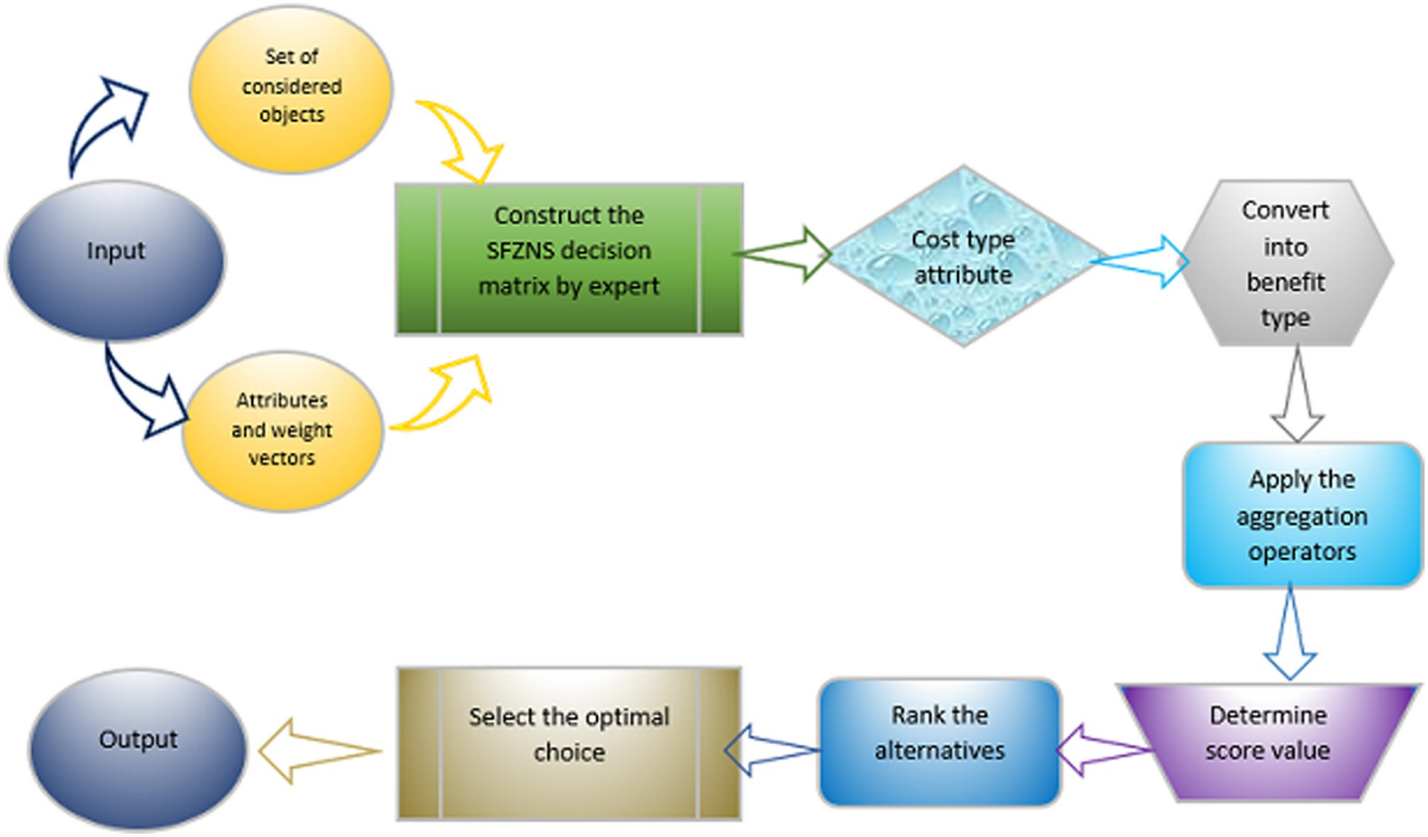
Flow chart of algorithm 1.

The flow chart of algorithm 1 is given in Fig 1:

## 6 Numerical example

The discussed methodology has been illustrated using a numerical example, the details of which are provided below. The suggested algorithms can rate how much an economic policy affects a certain province and pick the one that has the biggest effect.

In terms of economic development, several historical eras and nations have adopted various economic development techniques. National economic regulations and control policies are the key determinants of regional economic growth, influencing the pattern, pace, and quality of regional economic development under the direction of economic strategy. Economic regulation and control measures in a nation can take many different forms. Industrial policy, monetary policy, and fiscal policy have the greatest impact. In Table 2, each policy’s precise interpretation is displayed.

**Table 2:** Defines of attributes.

Attributes	Description
Industrial policy	Industrial strategy is a deliberate governmental effort to encourage economic transformation, or the move from lower to greater production activity, amongst or within sectors. Industrial policy isdescribed as “any type of selective government intervention or policy that tries to modify thestructure of production in favour of sectors that are regarded to provide greater opportunities foreconomic development in a manner that would not occur without such intervention in the marketequilibrium.”
Monetary policy	The objective of monetary policy is to maintain price stability and public trust in the current valueand stability of the nation’s currency. This is achieved through regulating the money supply or theinterest rate on very short-term borrowing, which refers to banks borrowing from one other tosatisfy their urgent needs.
Fiscal policy	Governments utilize fiscal policy, or spending restrictions, to stimulate the economy by adjusting thetax rates and expenditure allocations. Fiscal policy deals with taxes and public spending and isoften administered by a government agency, while monetary policy deals with interest rates and money supply and is typically administered by a country’s central bank.

For those seeking to identify and demonstrate the financial advantages, economic effect measurement has developed into a powerful and persuading instrument. The measuring impact takes into consideration the fact that numerous revenue and expenditure items answer to the state of the economy and Policymakers could better grasp how much their actions contribute to output stability by keeping track of the relationship between the budget balance and the output gap. This would allow them to compare their actions to those of other provinces and nations. How to evaluate the actual consequences of a nation’s economic policy on the local economy. We provide the following analysis strategy.

For this we suppose that *P* = {*p*_1_, *p*_2_, *p*_3_, *p*_4_} is the collection of four provinces **A**, **B**, **C**, and **D** of country **S** respectively and *W* = {*w*_1_, *w*_2_, *w*_3_} is the set of attributes indicating industrial policy, monetary policy, and fiscal policy respectively. Suppose λ = {λ_1_ = 0.38, λ_2_ = 0.47, λ_3_ = 0.15} is the attributes weight vectors and the corresponding associated weight vectors are *τ* = {0.3, 0.2, 0.5}. Then we get the best alternative (best province of country **S**) that has greatest impact of policies.

## 7 By using SF*Z̆*NWA and WG operator

**Step 1**: The information given by the expert in the SF*Z̆*Ns form is represented in the Table 3.

**Table 3:** Decision matrix by the expert.

*S*_*Qjv*_	*w*_1_	*w*_2_	*w*_3_
*p*_1_	((0.6,0.2),(0.3,0.4),(0.4,0.5))	((0.7,0.4),(0.4,0.4),(0.2,0.3))	((0.4,0.4),(0.2,0.6),(0.2,0.4))
*p*_2_	((0.4,0.3),(0.3,0.5),(0.2,0.3))	((0.6,0.7),(0.1,0.3),(0.2,0.5))	((0.4,0.5),(0.4,0.6),(0.2,0.4))
*p*_3_	((0.6,0.3),(0.2,0.6),(0.2,0.4))	((0.7,0.3),(0.2,0.5),(0.3,0.4))	((0.2,0.5),(0.3,0.4),(0.6,0.3))
*p*_4_	((0.7,0.3),(0.4,0.6),(0.5,0.4))	((0.6,0.4),(0.3,0.4),(0.4,0.6))	((0.4,0.3),(0.2,0.7),(0.3,0.5))

**Step 2**: Normalization is unnecessary due to the benefit-type nature of the criterion.

**Step 3**: Integrate the attributes for each alternative using SF*Z̆*NWA and WG operator, represented in Tables 4 and 5 respectively.

**Table 4:** Using SF*Z̆*NWA operator.

*S*_*Qj*_	*w*_*v*_
*p*_1_	((0.633113,0.340994),(0.323168,0.425083),(0.260268,0.38033))
*p*_2_	((0.510668,0.571517),(0.186902,0.404183),(0.2,0.39823))
*p*_3_	((0.623466,0.340611),(0.212541,0.51823),(0.285339,0.38311))
*p*_4_	((0.623268,0.351566),(0.314908,0.507493),(0.417009,0.50045))

**Table 5:** Using SF*Z̆*NWA operator.

*S*_*Qj*_	*w*_*v*_
*p*_1_	((0.60702,0.30738),(0.32317,0.42508),(0.2958,0.40566))
*p*_2_	((0.48397,0.48233),(0.1869,0.40418),(0.2,0.42305))
*p*_3_	((0.54708,0.32389),(0.21254,0.51823),(0.34507,0.38711))
*p*_4_	((0.59866,0.34343),(0.31491,0.50749),(0.43092,0.52273))

**Step 4**: Determine the score values.

By using SF*Z̆*NWA operator:

$$\begin{array}{c} ℑ\left(p_{1}\right)=0.65984,\;\;\;ℑ\left(p_{2}\right)=0.71222,\;\;\;ℑ\left(p_{3}\right)=0.6643,\;\;\;ℑ\left(p_{4}\right)=0.61687 \end{array}$$

By using SF*Z̆*NWG operator:

$$\begin{array}{c} ℑ\left(p_{1}\right)=0.64307,\;\;\;ℑ\left(p_{2}\right)=0.69109,\;\;\;ℑ\left(p_{3}\right)=0.64449,\;\;\;ℑ\left(p_{4}\right)=0.60684 \end{array}$$

**Step 5**: Choose the option that is most preferable after ranking all viable choices in descending order.

By using SF*Z̆*NWA operator:

$$\begin{array}{c} ℑ\left(p_{2}\right)>ℑ\left(p_{3}\right)>ℑ\left(p_{1}\right)>ℑ\left(p_{4}\right). \end{array}$$

By using SF*Z̆*NWG operator:

$$\begin{array}{c} ℑ\left(p_{2}\right)>ℑ\left(p_{3}\right)>ℑ\left(p_{1}\right)>ℑ\left(p_{4}\right). \end{array}$$

As a result, we determine that option *p*_2_ is the greatest choice.

## 8 By using SF*Z̆*NOWA and OWG operator

**Step 1**: The information given by the expert in the SF*Z̆*Ns form is represented in the Table 2.

**Step 2**: Normalization is unnecessary due to the benefit-type nature of the criterion.

**Step 3**: Evaluate the score value of each SF*Z̆*N and then re-order the SF*Z̆*N against each attribute as represented in Table 6.

**Table 6:** Re-ordered decision matrix.

*S*_*Qjv*_	*w*_*δ*(1)_	*w*_*δ*(2)_	*w*_*δ*(3)_
*p*_1_	((0.7,0.4),(0.4,0.4),(0.2,0.3))	((0.4,0.4),(0.2,0.6),(0.2,0.4))	((0.6,0.2),(0.3,0.4),(0.4,0.5))
*p*_2_	((0.6,0.7),(0.1,0.3),(0.2,0.5))	((0.4,0.3),(0.3,0.5),(0.2,0.3))	((0.4,0.5),(0.4,0.6),(0.2,0.4))
*p*_3_	((0.7,0.3),(0.2,0.5),(0.3,0.4))	((0.6,0.3),(0.2,0.6),(0.2,0.4))	((0.2,0.5),(0.3,0.4),(0.6,0.3))
*p*_4_	((0.6,0.4),(0.3,0.4),(0.4,0.6))	((0.4,0.3),(0.2,0.7),(0.3,0.5))	((0.7,0.3),(0.4,0.6),(0.5,0.4))

**Step 4**: Integrate the attributes for each alternative using SF*Z̆*NOWA and OWG operator, represented in Tables 7 and 8 respectively.

**Table 7:** Using SF*Z̆*NOWA operator.

*S*_*Qj*_	*w*_*δ*(*v*)_
*p*_1_	((0.57694,0.37816),(0.27659,0.48397),(0.22191,0.37078))
*p*_2_	((0.49241,0.53908),(0.20633,0.4232),(0.2,0.38033))
*p*_3_	((0.61326,0.34061),(0.21254,0.5268),(0.27511,0.38311))
*p*_4_	((0.54507,0.34244),(0.25888,0.55297),(0.36131,0.51823))

**Table 8:** Using SF*Z̆*NOWG operator.

*S*_*Qj*_	*w*_*δ*(*v*)_
*p*_1_	((0.52581,0.3605),(0.27659,0.48397),(0.24298,0.38556))
*p*_2_	((0.46663,0.44692),(0.20633,0.4232),(0.2,0.40566))
*p*_3_	((0.53954,0.32389),(0.21254,0.5268),(0.33886,0.38711))
*p*_4_	((0.50749,0.33466),(0.25888,0.55297),(0.37749,0.53081))

**Step 5**: Determine the score values.

By using SF*Z̆*NOWA operator:

$$\begin{array}{c} ℑ\left(p_{1}\right)=0.66734,\;\;\;Y\left(p_{2}\right)=0.70069,\;\;\;Y\left(p_{3}\right)=0.66384,\;\;\;Y\left(p_{4}\right)=0.61875 \end{array}$$

By using SF*Z̆*NOWG operator:

$$\begin{array}{c} ℑ\left(p_{1}\right)=0.654,\;\;\;Y\left(p_{2}\right)=0.68003,\;\;\;Y\left(p_{3}\right)=0.64387,\;\;\;Y\left(p_{4}\right)=0.60877 \end{array}$$

**Step 6**: Choose the option that is most preferable after ranking all viable choices in descending order.

By using SF*Z̆*NOWA operator:

$$\begin{array}{c} ℑ\left(p_{2}\right)>ℑ\left(p_{1}\right)>ℑ\left(p_{3}\right)>ℑ\left(p_{4}\right). \end{array}$$

By using SF*Z̆*NOWG operator:

$$\begin{array}{c} ℑ\left(p_{2}\right)>ℑ\left(p_{1}\right)>ℑ\left(p_{3}\right)>ℑ\left(p_{4}\right). \end{array}$$

As a result, we determine that option *p*_2_ is the greatest choice.

## 9 By using SF*Z̆*NHA and HG operator

**Step 1**: The information given by the expert in the SF*Z̆*Ns form is represented in the Table 3.

**Step 2**: Normalization is unnecessary due to the benefit-type nature of the criterion.

**Step 3**: Evaluate the weighted values matrix using *τ*_*v*_ weighted vectors, represented in Tables 9 and 10.

**Table 9:** Weighted values decision matrix using HA operator.

$S_{Qjv}^{\prime }$	*w*_*δ*(1)_	*w*_*δ*(2)_
*p*_1_	((0.63148,0.21324),(0.25347,0.35184),(0.35184,0.45376))	((0.78296,0.46685),(0.27473,0.27473),(0.10338,0.18312))
*p*_2_	((0.42457,0.31927),(0.25347,0.45376),(0.15965,0.25347))	((0.68339,0.78296),(0.0389,0.18312),(0.10338,0.37631))
*p*_3_	((0.63148,0.31927),(0.15965,0.55859),(0.15965,0.35184))	((0.78296,0.35287),(0.10338,0.37631),(0.18312,0.27473))
*p*_4_	((0.73204,0.31927),(0.35184,0.55859),(0.45376,0.35184))	((0.68339,0.46685),(0.18312,0.27473),(0.27473,0.48662))
$S_{Qjv}^{\prime }$	*w*_*δ*(3)_	
*p*_1_	((0.2747,0.2747),(0.48469,0.79464),(0.48469,0.66211))	
*p*_2_	((0.2747,0.34846),(0.66211,0.79464),(0.484569,0.66211))	
*p*_3_	((0.13492,0.34846),(0.58171,0.66211),(0.79464,0.58171))	
*p*_4_	((0.2747,0.20384),(0.48469,0.85171),(0.58171,0.73204))	

**Table 10:** Weighted values decision matrix using HG operator.

$S_{Qjv}^{\prime }$	*w*_*δ*(1)_	*w*_*δ*(2)_
*p*_1_	((0.55859,0.15965),(0.25347,0.35184),(0.42457,0.52878))	((0.60477,0.27473),(0.27473,0.27473),(0.2365,0.35287))
*p*_2_	((0.35184,0.25347),(0.25347,0.45376),(0.21324,0.31927))	((0.48662,0.60477),(0.0389,0.18312),(0.2365,0.57745))
*p*_3_	((0.55859,0.25347),(0.15965,0.55859),(0.21324,0.42457))	((0.60477,0.18312),(0.10338,0.37631),(0.35287,0.46685))
*p*_4_	((0.6659,0.25347),(0.35184,0.55859),(0.52878,0.42457))	((0.48662,0.27473),(0.18312,0.27473),(0.46685,0.68339))
$S_{Qjv}^{\prime }$	*w*_*δ*(3)_	
*p*_1_	((0.66211,0.66211),(0.48469,0.79464),(0.13492,0.2747))	
*p*_2_	((0.66211,0.73204),(0.66211,0.79464),(0.13492,0.2747))	
*p*_3_	((0.48469,0.73204),(0.58171,0.66211),(0.42655,0.20384))	
*p*_4_	((0.66211,0.58171),(0.48469,0.85171),(0.20384,0.34846))	

**Step 4**: Evaluate the score value of each SF*Z̆*N weighted values and then re-order the SF*Z̆*N against each attribute as represented in Tables 11 and 12.

**Table 11:** Re-ordered decision matrix by HA operator.

$S_{Qjv}^{\prime }$	*w*_*δ*(1)_	*w*_*δ*(2)_
*p*_1_	((0.78296,0.46685),(0.27473,0.27473),(0.10338,0.18312))	((0.63148,0.21324),(0.25347,0.35184),(0.35184,0.45376))
*p*_2_	((0.68339,0.78296),(0.0389,0.18312),(0.10338,0.37631))	((0.42457,0.31927),(0.25347,0.45376),(0.15965,0.25347))
*p*_3_	((0.78296,0.35287),(0.10338,0.37631),(0.18312,0.27473))	((0.63148,0.31927),(0.15965,0.55859),(0.15965,0.35184))
*p*_4_	((0.68339,0.46685),(0.18312,0.27473),(0.27473,0.48662))	((0.73204,0.31927),(0.35184,0.55859),(0.45376,0.35184))
$S_{Qjv}^{\prime }$	*w*_*δ*(3)_	
*p*_1_	((0.2747,0.2747),(0.48469,0.79464),(0.48469,0.66211))	
*p*_2_	((0.2747,0.34846),(0.66211,0.79464),(0.48469,0.66211))	
*p*_3_	((0.13492,0.34846),(0.58171,0.66211),(0.79464,0.58171))	
*p*_4_	((0.2747,0.20384),(0.48469,0.85171),(0.58171,0.73204))	

**Table 12:** Re-ordered decision matrix by HG operator.

$S_{Qjv}^{\prime }$	*w*_*δ*(1)_	*w*_*δ*(2)_
*p*_1_	((0.66211,0.66211),(0.48469,0.79464),(0.13492,0.2747))	((0.60477,0.27473),(0.27473,0.27473),(0.2365,0.35287))
*p*_2_	((0.48662,0.60477),(0.0389,0.18312),(0.2365,0.57745))	((0.66211,0.73204),(0.66211,0.79464),(0.13492,0.2747))
*p*_3_	((0.55859,0.25347),(0.15965,0.55859),(0.21324,0.42457))	((0.60477,0.18312),(0.10338,0.37631),(0.35287,0.46685))
*p*_4_	((0.66211,0.58171),(0.48469,0.85171),(0.20384,0.34846))	((0.48662,0.27473),(0.18312,0.27473),(0.46685,0.68339))
$S_{Qjv}^{\prime }$	*w*_*δ*(3)_	
*p*_1_	((0.55859,0.15965),(0.25347,0.35184),(0.42457,0.52878))	
*p*_2_	((0.35184,0.25347),(0.25347,0.45376),(0.21324,0.31927))	
*p*_3_	((0.48469,0.73204),(0.58171,0.66211),(0.42655,0.20384))	
*p*_4_	((0.6659,0.25347),(0.35184,0.55859),(0.52878,0.42457))	

**Step 5**: Integrate the attributes for each alternative using SF*Z̆*NHA and HG operator, represented in Tables 13 and 14 respectively.

**Table 13:** Using SF*Z̆*NHA operator.

*S*_*Qj*_	$w_{\delta (v)}^{\prime }$
*p*_1_	((0.58886,0.33927),(0.35908,0.49094),(0.28598,0.41749))
*p*_2_	((0.48467,0.55677),(0.23348,0.45737),(0.24418,0.46122))
*p*_3_	((0.57169,0.34423),(0.2675,0.54019),(0.37114,0.42004))
*p*_4_	((0.56319,0.33158),(0.33949,0.55749),(0.44197,0.55937))

**Table 14:** Using SF*Z̆*NHG operator.

*S*_*Qj*_	$w_{\delta (v)}^{\prime }$
*p*_1_	((0.59723,0.27268),(0.31288,0.42757),(0.33149,0.43902))
*p*_2_	((0.44007,0.40676),(0.17504,0.38661),(0.20796,0.41711))
*p*_3_	((0.52866,0.40364),(0.27938,0.56195),(0.36261,0.34917))
*p*_4_	((0.62434,0.33048),(0.33991,0.55007),(0.44965,0.48175))

**Step 6**: Determine the score values.

By using SF*Z̆*NHA operator:

$$\begin{array}{c} ℑ\left(p_{1}\right)=0.6347,\;\;\;ℑ\left(p_{2}\right)=0.68348,\;\;\;ℑ\left(p_{3}\right)=0.63213,\;\;\;ℑ\left(p_{4}\right)=0.58342 \end{array}$$

By using SF*Z̆*NHG operator:

$$\begin{array}{c} ℑ\left(p_{1}\right)=0.62785,\;\;\;ℑ\left(p_{2}\right)=0.67486,\;\;\;ℑ\left(p_{3}\right)=0.64326,\;\;\;ℑ\left(p_{4}\right)=0.60092 \end{array}$$

**Step 7**: Choose the option that is most preferable after ranking all viable choices in descending order.

By using SF*Z̆*NHA operator:

$$\begin{array}{c} ℑ\left(p_{2}\right)>ℑ\left(p_{1}\right)>ℑ\left(p_{3}\right)>ℑ\left(p_{4}\right). \end{array}$$

By using SF*Z̆*NHG operator:

$$\begin{array}{c} ℑ\left(p_{2}\right)>ℑ\left(p_{3}\right)>ℑ\left(p_{1}\right)>ℑ\left(p_{4}\right). \end{array}$$

As a result, we determine that option *p*_2_ is the greatest choice.

## 10 TODIM approach for SF*Z̆*Ns

To effectively deal with MCDM challenges, Gomes [37] created the TODIM technique in 1990, which is based on prospect theory. The TODIM technique is a type of interactive MCDM that uses prospect theory and takes into account the psychological traits of the people making the decisions. We choose to use the TODIM technique in a SF*Z̆*Ns setting to solve this challenge and enhance the rationality of the decisions in light of the aforementioned studies. The TODIM method is a multi-criteria decision-making approach that can be used to evaluate and rank alternatives based on multiple criteria. It is particularly useful when the criteria are not easily quantifiable and can be subjective. The TODIM method involves the use of triangular fuzzy numbers to represent the criteria and the alternatives, and a weighting system to determine the relative importance of each criterion. By using this method, decision-makers can make informed decisions that take into account multiple factors and their relative importance, leading to more accurate and well-rounded choices.

Suppose the collection of alternatives *P* = {*p*_1_, *p*_2_, *p*_3_…, *p*_*m*_} and the set of attributes *W* = {*w*_1_, *w*_2_, …, *w*_*g*_} with the weight vectors λ = {λ_1_, λ_2_, …, λ_*g*_}. The weight vector requirement is that weights must belong to a closed unit interval and that their sum must be equal to 1. Then, based on the suggested aggregation operators, we summarized the subsequent steps to determine the best solution among the feasible ones.

**Algorithm 6.1**

***Step 1**: Consider universal set, weight vectors and attribute’s set as an input then construct the SFZ̆Ns decision matrix as follows S*_*Q*_ = [*S*_*Qjv*_]_*m*×*g*_ *after collecting expert evaluation information regarding each alternative’s attributes*.

***Step 2**: There are two different types of criteria used widely, one of which is referred to as a positive criterion and the other as a negative criterion. We must change the negative criteria into positive criteria by taking the complement for the negative criterion.*

***Step 3**: Identify each attribute’s relative weight vectors*:

$$\begin{array}{c} \lambda_{vq}=\frac{\lambda_{v}}{\lambda_{q}}\;;v=1,2,...,g \end{array}$$

*where* λ_*v*_ *is the weight vector of attribute* (*w*_*v*_) *and* λ_*q*_ = *max*{λ_1_, λ_2_, …, λ_*g*_}.

***Step 4**: Evaluate the dominance degree of each alternative over each other alternative*.

$$\begin{array}{c} \;\phi_{v}\left(p_{j},p_{k}\right)=\{\begin{array}{cc} \;\sqrt{\lambda_{vq}d(ℑ\left(S_{Qjv}\right),ℑ\left(S_{Qkv}\right))/\sum_{v=1}^{g}\lambda_{vq}} & ,\;\mathrm{if}\;\;ℑ\left(S_{Qjv}\right)>ℑ\left(S_{Qkv}\right),\\ 0 & ,\;\mathrm{if}\;\;ℑ\left(S_{Qjv}\right)=ℑ\left(S_{Qkv}\right),\\ -\frac{1}{\vartheta }\sqrt{(\sum_{v=1}^{g}\lambda_{vq})d(ℑ\left(S_{Qkv}\right),ℑ\left(S_{Qjv}\right))/\lambda_{vq}} & ,\;\mathrm{if}\;\;ℑ\left(S_{Qjv}\right)<ℑ\left(S_{Qkv}\right). \end{array} \end{array}$$

*where the parameter ϑ* > 0 *illustrate the attenuation component of the losses, the distance d*(ℑ(*S*_*Qjv*_), ℑ(*S*_*Qkv*_)) *can be estimated using definition 3.6 and* ℑ(*S*_*Qjv*_) *can be obtained by using definition 3.3 and 3.4*.

***Step 5**: Determine the overall dominance degree of each alternative by*:

$$\begin{array}{c} \varphi \left(p_{j},p_{k}\right)=\sum_{v=1}^{g}\phi_{v}\left(p_{j},p_{k}\right),\;\;\forall \left(j,k\right), \end{array}$$

***Step 6**: Evaluate the overall value of each alternative p*_*j*_ *by*:

$$\begin{array}{c} S\left(p_{j}\right)=\frac{\sum_{k=1}^{m}\phi_{v}\left(p_{j},p_{k}\right)-\min_{v=1}^{g}\left\{\sum_{k=1}^{m}\phi_{v}\left(p_{j},p_{k}\right)\right\}}{\max_{v=1}^{g}\left\{\sum_{k=1}^{m}\phi_{v}\left(p_{j},p_{k}\right)\right\}-\min_{v=1}^{g}\left\{\sum_{k=1}^{m}\phi_{v}\left(p_{j},p_{k}\right)\right\}}. \end{array}$$

***Step 7**: Rank the all alternatives in descending order and choose the best one*.

**Fig 2:**
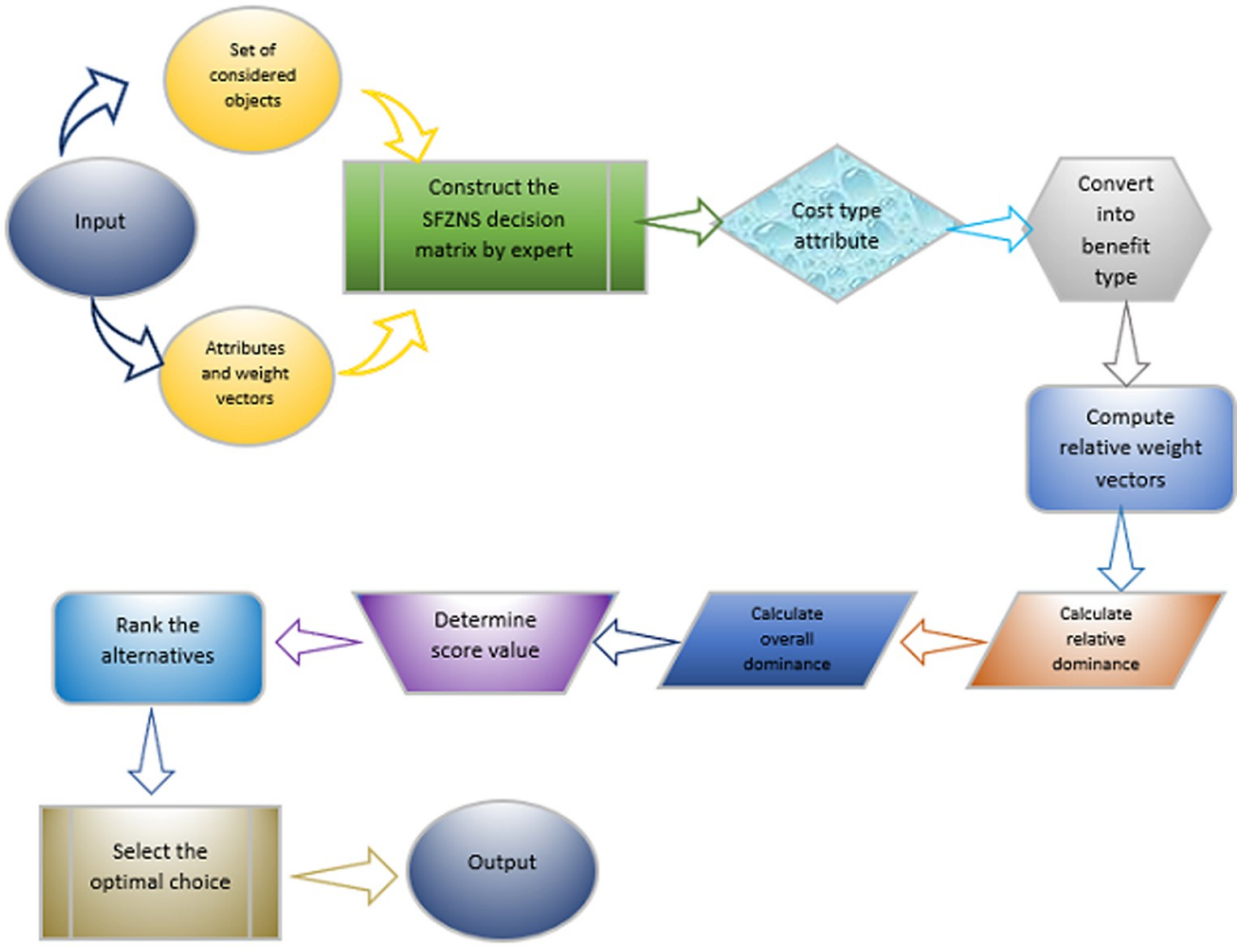
Flow chart of algorithm 2.

Fig 2 shows the flowchart of algorithm 2:

## 11 Numerical illustration

We will reconsider the case study described in the previous section to demonstrating the applicability and efficacy of our suggested TODIM algorithms in the MCDM context is provided in this section.

**Step 1**: The information given by the expert in the SF*Z̆*Ns form is represented in the Table 3.

**Step 2**: Normalization is unnecessary due to the benefit-type nature of the criterion.

**Step 3**: Compute relative weight vectors for each attribute.

$$\begin{array}{c} \lambda_{1q}=\frac{\lambda_{1}}{\lambda q}=\frac{0.38}{0.47}=0.80851,\;\;\;\;\lambda_{2q}=\frac{\lambda_{2}}{\lambda_{q}}=\frac{0.47}{0.47}=1,\;\;\;\;\lambda_{3q}=\frac{\lambda_{3}}{\lambda_{q}}=\frac{0.15}{0.47}=0.31915. \end{array}$$

**Step 4**: Obtain the relative dominance of each alternative as represented in the Tables 15–17 and *ϑ* = 1.

**Table 15:** Dominance degree relative to *w*_1_.

*ϕ*_1_(*p*_*j*_, *p*_*k*_)	*p*_1_	*p*_2_	*p*_3_	*p*_4_
*p*_1_	0	-0.61383	-0.59419	0.23875
*p*_2_	0.23325	0	-0.4292	0.26814
*p*_3_	0.22579	0.1631	0	0.25591
*p*_4_	-0.62828	-0.70563	-0.67344	0

**Table 16:** Dominance degree relative to *w*_2_.

*ϕ*_2_(*p*_*j*_, *p*_*k*_)	*p*_1_	*p*_2_	*p*_3_	*p*_4_
*p*_1_	0	-0.64444	0.22738	0.29779
*p*_2_	0.30289	0	0.32321	0.33944
*p*_3_	-0.48378	-0.68767	0	0.24267
*p*_4_	-0.6336	-0.72222	-0.51633	0

**Table 17:** Dominance degree relative to *w*_3_.

*ϕ*_3_(*p*_*j*_, *p*_*k*_)	*p*_1_	*p*_2_	*p*_3_	*p*_4_
*p*_1_	0	0.13774	0.13226	0.11162
*p*_2_	-0.9183	0	-0.1668	0.14796
*p*_3_	-0.88174	-1.11197	0	-0.52428
*p*_4_	-0.74416	-0.98639	0.07864	0

**Step 5**: Obtain overall dominance of the each alternative, as represented in the Table 18.

**Table 18:** Overall dominance degree.

*φ*(*p*_*j*_, *p*_*k*_)	*p*_1_	*p*_2_	*p*_3_	*p*_4_
*p*_1_	0	-1.12052	-0.23455	0.64816
*p*_2_	-0.38216	0	0.06081	0.75554
*p*_3_	-1.13972	-1.63655	0	-0.0257
*p*_4_	-2.00604	-2.41424	-1.11112	0

**Step 6**: Determine the overall total value of each option.

$$\begin{array}{c} S\left(p_{1}\right)=0.80872,\;\;\;S\left(p_{2}\right)=1,\;\;\;S\left(p_{3}\right)=0.45753,\;\;\;S\left(p_{4}\right)=0. \end{array}$$

**Step 7**: Choose the option that is most preferable after ranking all viable choices in descending order.

$$\begin{array}{c} S\left(p_{2}\right)>S\left(p_{1}\right)>S\left(p_{3}\right)>S\left(p_{4}\right). \end{array}$$

As a result, we determine that option *p*_2_ is the greatest choice.

## 12 Relative comparison

**Fig 3:**
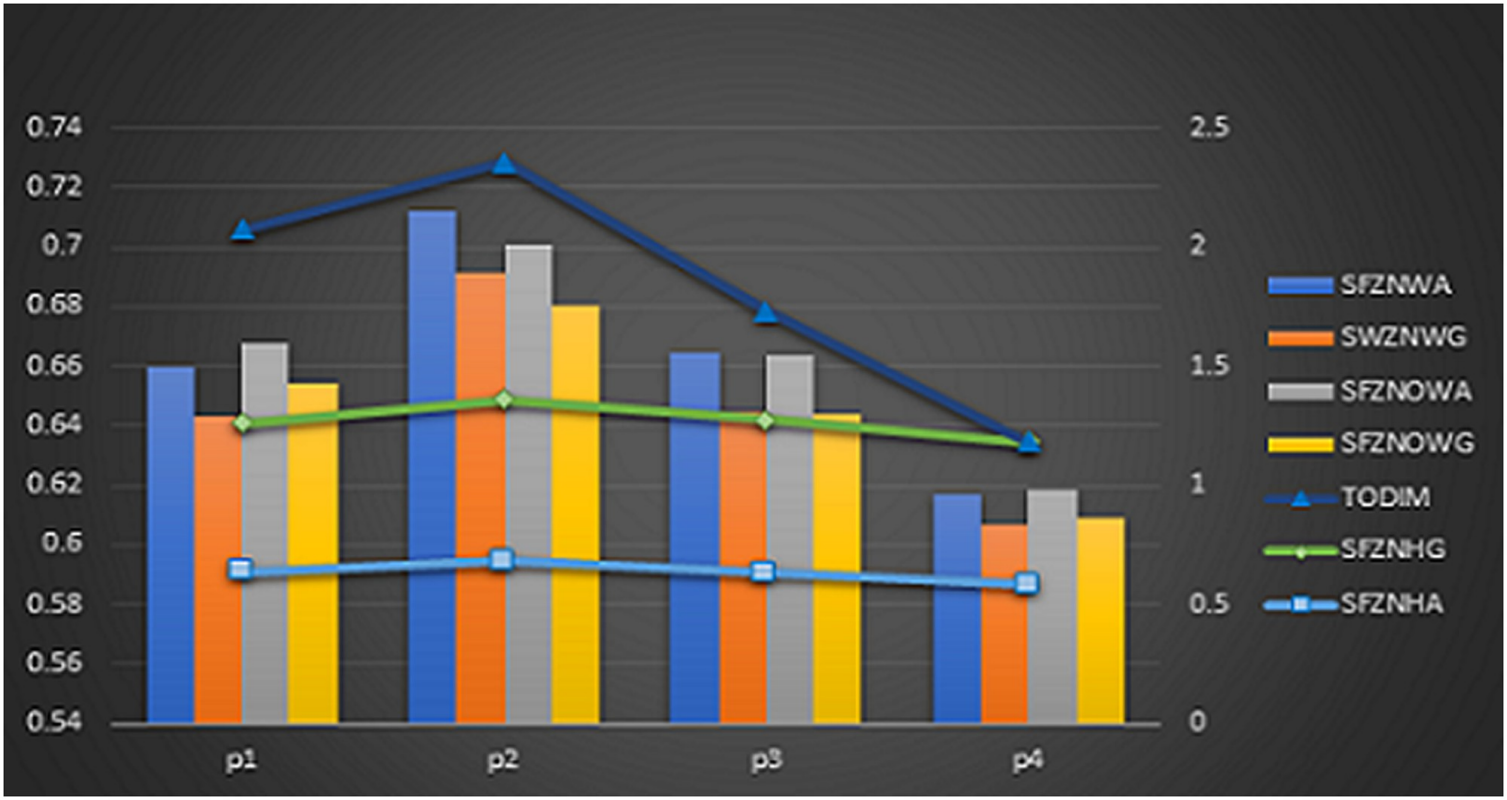
Graphical representation of ranking.

An observation has been carried out to compare the effectiveness of the proposed algorithms with some of the current measures in the spherical fuzzy *Z̆*-numbers. The ranking order is little bit different but the optimal choice is same in all approaches. The ranking and graphical representation of all the operators such as SF*Z̆*NWA, SF*Z̆*NOWA, SF*Z̆*NHA, SF*Z̆*NWG, SF*Z̆*NOWG, and SF*Z̆*NHG and TODIM approach is given in the Table 19 and Fig 3:

**Table 19:** Ranking using various operator and TODIM approach.

Operators	Final Ranking
SF*Z̆*NWA	*p*_2_ > *p*_3_ > *p*_1_ > *p*_4_
SF*Z̆*NWG	*p*_2_ > *p*_3_ > *p*_1_ > *p*_4_
SF*Z̆*NOWA	*p*_2_ > *p*_1_ > *p*_3_ > *p*_4_
SF*Z̆*NOWG	*p*_2_ > *p*_1_ > *p*_3_ > *p*_4_
SF*Z̆*NHA	*p*_2_ > *p*_1_ > *p*_3_ > *p*_4_
SF*Z̆*NHG	*p*_2_ > *p*_3_ > *p*_1_ > *p*_4_
TODIM	*p*_2_ > *p*_1_ > *p*_3_ > *p*_4_

The weighted SF*Z̆*Ns averaging operator simply takes into account the significance of the aggregated spherical fuzzy *Z̆*-numbers sets themselves. The SF*Z̆*NOWA operator solely considers the ranking order of the aggregated spherical fuzzy sets and its position’s importance. While the SF*Z̆*Ns hybrid weighted aggregation operator consider the both properties of weighted and order weighted operator simultaneously. The suggested approach is more effective since the decision-maker(s) can choose the characteristics and operators based on their specific needs and prevailing conditions.

## 13 Discussion

It is clear from the results that the ranking orders can be impacted by MADM approaches using different decision information. The improved MADM approach makes use of hybrid evaluation data from both spherical values and spherical measures of comparable dependability, such that the existing spherical MADM method use only single-valued assessment data spherical value and takes the relevant dependability metrics into consideration. This is the reason why the results rank differently. The proposed MADM technique makes use of the combined assessment data of spherical numbers and sphere measurements of matching reliability, such that the prevailing spherical MADM method solely uses spherical measures. The reliability metrics that have been incorporated undoubtedly improve the information representation and credibility of the assessment process as well as the ranked order of choices, demonstrating the efficacy and logic of the created MADM strategy. SF*Z̆*Ns is an uncertain and inconsistent environment, reliability measures linked to spherical data enrich the measure data of durability because they demonstrate a stronger capability to express human knowledge and judgments using spherical values, and durability potential involve spherical values. Thus, in the MADM problem, the informative presentation of SF*Z̆*N is preferable to that of one individual spherical score and only one *Z̆*-number. As a result, the new MADM methodology provided in this study provides a more broad version of MADM theory and procedure recognition.The new MADM technique may fix the problems with the spherical MADM methodology and improve MADM reliability and efficacy, which exhibit the most notable benefits.

The advantage of using spherical fuzzy *Z̆*-numbers over traditional fuzzy *Z̆*-numbers is that they provide a more flexible and accurate representation of uncertain information in multidimensional space. Specifically, spherical fuzzy *Z̆*-numbers can capture uncertainty in both the magnitude and direction of a quantity, whereas traditional fuzzy numbers and fuzzy *Z̆*-numbers only capture uncertainty in magnitude.They can be useful in various areas such as:

1. Decision-making: Spherical fuzzy *Z̆*-numbers can be used in multi-criteria decision-making (MCDM) problems to handle imprecise, uncertain, and incomplete information.
2. Risk analysis: Spherical fuzzy *Z̆*-numbers can be used in risk analysis to model and analyze the uncertainty associated with risk events.
3. Engineering: Spherical fuzzy *Z̆*-numbers can be used in engineering problems, such as in the design of robust systems or in the analysis of complex systems with multiple sources of uncertainty.
4. Finance: Spherical fuzzy *Z̆*-numbers can be used in finance to model and analyze financial data, such as stock prices or exchange rates, which are often characterized by uncertainty and imprecision.
5. Medical diagnosis: Spherical fuzzy *Z̆*-numbers can be used in medical diagnosis to model and analyze the uncertainty associated with diagnostic tests or patient data.

## 14 Conclusion

This study’s main objective is to develop fundamental operational rules for SF*Z̆*Ns employing aggregation operators. Following that, new operators like SF*Z̆*NWA, SF*Z̆*NOWA, SF*Z̆*NHA, SF*Z̆*NWG, SF*Z̆*NOWG, and SF*Z̆*NHG are developed using the designed operational laws. Numerous fundamental features, theorems, and properties are also presented for the proposed aggregation operators. To address MADM difficulties, a DM strategy based on endorsed operators and the TODIM strategy has been created, which incorporates the parameter into the calculation process to take both the positive and negative elements into account when making decisions. However, the key flaw of conventional TODIM is its inability to deal with ambiguity and incomplete information while making decisions. Also, it still has a few flaws, though, like non-discriminatory and counter-intuitive issues. Therefore, spherical fuzzy sets and fuzzy *Z̆*-numbers will apply with traditional TODIM to address this weakness. We also provide a detailed mathematical illustration. Finally, based on the results, it is determined that the approach suggested in this study is the most beneficial and effective strategy to handle the MADM issue. The focus of future research will be on developing novel decision-making methods for the SF*Z̆*Ns scenario that make use of various operators, such as the generalized geometric and average operators suggested by Einstein and Frank as well as EDAS and electric method to enhance the efficacy of DM.

### Future of work

Here are some potential areas of future research on spherical fuzzy *Z̆* numbers:

1. Mathematical properties of SF*Z̆*: Further investigation into the mathematical properties of SF*Z̆*, such as algebraic operations, aggregation operators, and distance measures, is necessary.
2. Applications of SF*Z̆* in decision-making: SF*Z̆* can be applied in a wide range of decision-making problems, including multi-criteria decision-making, fuzzy decision-making, and risk assessment. Future research could explore the use of SF*Z̆* in these and other decision-making contexts.
3. Hybrid models: Hybrid models that combine SF*Z̆* with other techniques, such as artificial neural networks, genetic algorithms, and rough sets, could be developed to improve the accuracy of decision-making models.
4. Optimization algorithms:Development of optimization algorithms for SF*Z̆*-based decision-making problems is another potential area of future research.
5. Real-world applications: Finally, there is a need for empirical studies that apply SF*Z̆* in real-world decision-making problems.
